# The flow of axonal information among hippocampal sub-regions 2: patterned stimulation sharpens routing of information transmission

**DOI:** 10.3389/fncir.2023.1272925

**Published:** 2023-12-08

**Authors:** Samuel Brandon Lassers, Yash S. Vakilna, William C. Tang, Gregory J. Brewer

**Affiliations:** ^1^Department of Biomedical Engineering, University of California, Irvine, Irvine, CA, United States; ^2^Texas Institute of Restorative Neurotechnologies (TIRN), The University of Texas Health Science Center (UTHealth), Houston, TX, United States; ^3^Department of Biomedical Engineering, National Taiwan University, Taipei, Taiwan; ^4^Memory Impairments and Neurological Disorders (MIND) Institute, Center for Neuroscience of Learning and Memory, University of California, Irvine, Irvine, CA, United States

**Keywords:** networks, hippocampus, electrode array, entorhinal, dentate, CA3, CA1, neural circuits

## Abstract

The sub-regions of the hippocampal formation are essential for episodic learning and memory formation, yet the spike dynamics of each region contributing to this function are poorly understood, in part because of a lack of access to the inter-regional communicating axons. Here, we reconstructed hippocampal networks confined to four subcompartments in 2D cultures on a multi-electrode array that monitors individual communicating axons. In our novel device, somal, and axonal activity was measured simultaneously with the ability to ascertain the direction and speed of information transmission. Each sub-region and inter-regional axons had unique power-law spiking dynamics, indicating differences in computational functions, with abundant axonal feedback. After stimulation, spiking, and burst rates decreased in all sub-regions, spikes per burst generally decreased, intraburst spike rates increased, and burst duration decreased, which were specific for each sub-region. These changes in spiking dynamics post-stimulation were found to occupy a narrow range, consistent with the maintenance of the network at a critical state. Functional connections between the sub-region neurons and communicating axons in our device revealed homeostatic network routing strategies post-stimulation in which spontaneous feedback activity was selectively decreased and balanced by decreased feed-forward activity. Post-stimulation, the number of functional connections per array decreased, but the reliability of those connections increased. The networks maintained a balance in spiking and bursting dynamics in response to stimulation and sharpened network routing. These plastic characteristics of the network revealed the dynamic architecture of hippocampal computations in response to stimulation by selective routing on a spatiotemporal scale in single axons.

## Introduction

The function of the trisynaptic loop in the mammalian hippocampus is widely understood to govern the processes of learning and episodic memory. However, the spike dynamics that support the specific functions of each sub-region of this network have not been compared in a unitary system. The trisynaptic loop is composed of distinct sub-regions that each have a unique computational role in integrating and processing information transmitted from the neocortex to the hippocampus. Current computational models of hippocampal architecture suggest that the trisynaptic loop allows episodes to be completed from only partial cues, enables spatial learning through arbitrary associations, and is able to complete and associate learned and novel patterns (Kesner and Rolls, 2015). From a systems-level perspective, the hippocampus integrates sensory information, higher level computations from the prefrontal cortex, amygdala, orbitofrontal cortex, and the entorhinal cortex (EC), allowing for further computation of object and spatial representations and reward-related information (Kesner and Rolls, 2015). Information from the EC is routed to the dentate gyrus (DG) along the perforant pathway where pattern separation is proposed to occur. Sparse but powerful mossy fiber connections project unique representations to the CA3 for learning features. The CA3 associates information types, rapidly encodes new information, and facilitates recall. Information from the CA3 is sequenced and ordered in the CA1 for a coherent experience, which then routes information back to the EC for back projection into the neocortex. While these general functions of hippocampal sub-regions are known, the spiking dynamics that encode these functions within the sub-region and how that information is communicated to other sub-regions through axons are not well understood. Specifically, how the spiking dynamics of the sub-regions of the trisynaptic loop represent information computation at a system level is poorly understood as many studies focus on one or at most two sub-regions.

### Excitatory and inhibitory balance of hippocampal activity

Neural networks maintain a delicate balance between excitatory (E) and inhibitory (I) balance where spontaneous firing frequency is maintained within a tight range (Maffei and Fontanini, 2009). Dysregulation of this balance for extended periods of time is associated with neurological pathologies, including autism, schizophrenia, and Alzheimer's disease (He and Cline, 2019; Sohal and Rubenstein, 2019; Markicevic et al., 2020; Molina et al., 2020). Definitions of E/I balance range from the global balance of a relatively constant ratio of contributions of excitation and inhibition over a long-time scale to a short-time scale where E/I magnitudes are relatively well matched. Whether this E/I balance of spiking dynamics in response to stimulation is maintained within each sub-region or only over the whole hippocampal network is not well studied.

### Stimulation of neural cells

Behavioral studies of memory in animal models rely on complex inputs from environmental factors that usually build on anthropomorphic psychologies. These undefined input signals in combination with differing psychologies between humans and animals have led to a failure of behavioral studies to predict human responses (Lynch and Gall, 2013). Stimulation of cells is necessary in order to understand functional relationships. Electrical stimulation provides a known signal input for to study global cell dynamics at the expense of knowing the behavioral meaning behind the cellular outputs. Theta burst stimulation is a common electrical stimulation applied to neuronal tissue comprising 20 to 40 pulses at high frequency (100 Hz), repeated at theta frequencies of 4–9 Hz that mimics activities during learning. This stimulation paradigm evokes long-term potentiation in many elements of the network, a process that strengthens synapses between neurons lasting for months (Lynch and Gall, 2013). Characterization of network dynamics under different theta burst stimulation protocols activates different molecular mechanisms of LTP underpinning learning (Zhu et al., 2015).

### Novelty

With our microelectromechanical systems (MEMS) device, we simultaneously recorded spiking activity in the four sub-regions of the hippocampus and the axons connecting the sub-regions. With two electrodes under axons between sub-regions, the directionality of information flow could be measured, and the balance of sub-regional information could be contextualized with axonal control mechanisms. This enabled the measurement of local and global balance between feed-forward and feedback activity. Additionally, functional connectivity analysis was ascertained between the axons and soma to determine the directional effects axons have on sub-regional spiking activity and vice versa. As the brain functions probabilistically, the reliability of functional connectivity could inform routing strategies the network uses in order to balance E/I activity, which underpins the dynamics of information processing. Culturing and stimulating a live network on an MEA provide knowledge of the input stimulus, which is unknown in whole animal models. Shortly after our networks were stimulated, spontaneous activity was recorded for 5 min so that homeostatic and information-processing states could be compared.

## Hypothesis

**Figure 1:**
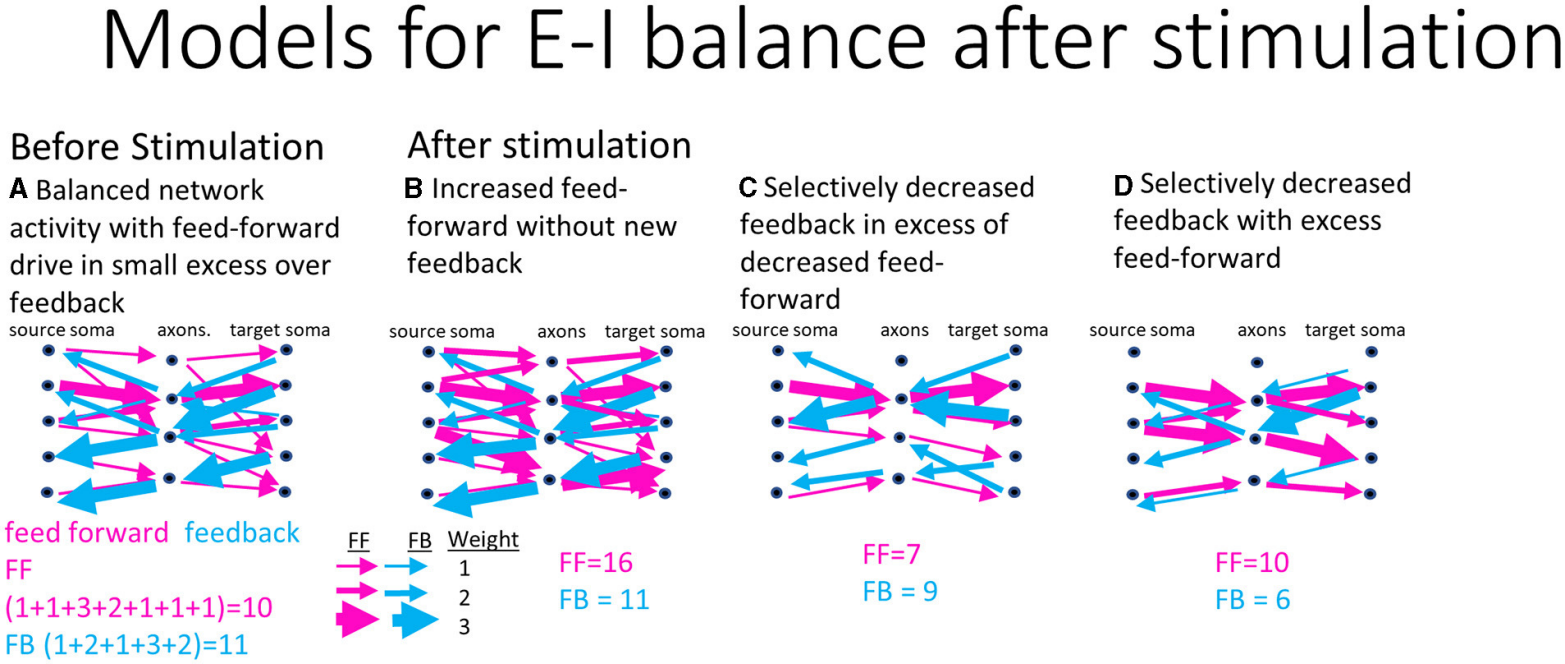
Possible models for excitatory and inhibitory routing balance after stimulation of a source somal layer, through communicating axons into target somata in hippocampal networks. Weighting of example connections are indicated by the thickness of arrows, feed-forward (red), and feedback (blue). The sum of their activity in each case is indicated. **(A)** Modeled homeostatic conditions before any stimulation input. **(B)** Predicted routing model after stimulation shows an increase in feed-forward routing and maintenance of feedback routing for overall increased activity. **(C)** Second predicted model of routing with fewer feedback routes in excess of decreased feed-forward routes for a net decreased modulation of activity. **(D)** Selectively decreased feed-forward in excess of decreased feedback routing for overall decreased activity.

Cultured cortical neural networks reach homeostatic conditions of balanced excitatory and inhibitory activity when left to grow for weeks unperturbed (Beggs, 2022). After electrical stimulation, the network is pushed away from these homeostatic conditions and must respond by rebalancing excitatory and inhibitory activity. This requires changing the spiking dynamics and routing strategies that function as a control system for computing new information. As a control, in a two-compartment system, Brewer et al. (2013) found specific spike dynamics in DG-CA3 compared to DG-DG and CA3-CA3 suggesting that appropriate anatomy produced distinct coding. Figure 1 illustrates three model possibilities for the network to balance excitatory and inhibitory activity after stimulation. Figure 1A proposes the starting, before stimulation, functional balance of connections between a network sub-region and its axonal tunnels under balanced homeostatic excitatory feed-forward and inhibitory feedback activity. Figures 1B–D proposes different strategies the network might use to route information to restore network balance after stimulation. Figure 1B proposes more excitatory routes are recruited while the same feedback routes remain after stimulation for overall increased activity. This suggests the network at homeostasis is not maximally computing information and has the capacity to carry more computational load. Figure 1C suggests that feedback activity quickly dampens the feed-forward activity after stimulation as a control mechanism for an overall decrease in activity. This could select specific routing to process new information. Figure 1D suggests that feed-forward routing remains but feedback routing decreased for an increased dominance of feed-forward activity. This would be similar to Figure 1B in suggesting that the hippocampus takes on more load in response to stimulation but by decreased inhibition. The null hypothesis is that feed-forward and feedback responses remain at the same levels and balance. We do not expect the responses to increase proportionally as that would require increased spike rates that we and others have already shown to decrease after electrical stimulation (Wagenaar et al., 2006; Chen et al., 2023). Using data from a reverse-engineered hippocampus, our goal was to discriminate between these possible models to better understand the strategies of computation in the hippocampus and associated spiking dynamics in response to stimulation. This will help contextualize the relative computational contributions of each sub-region by accessing interregional axonal communication.

## Methods

### Prior efforts

**Figure 2:**
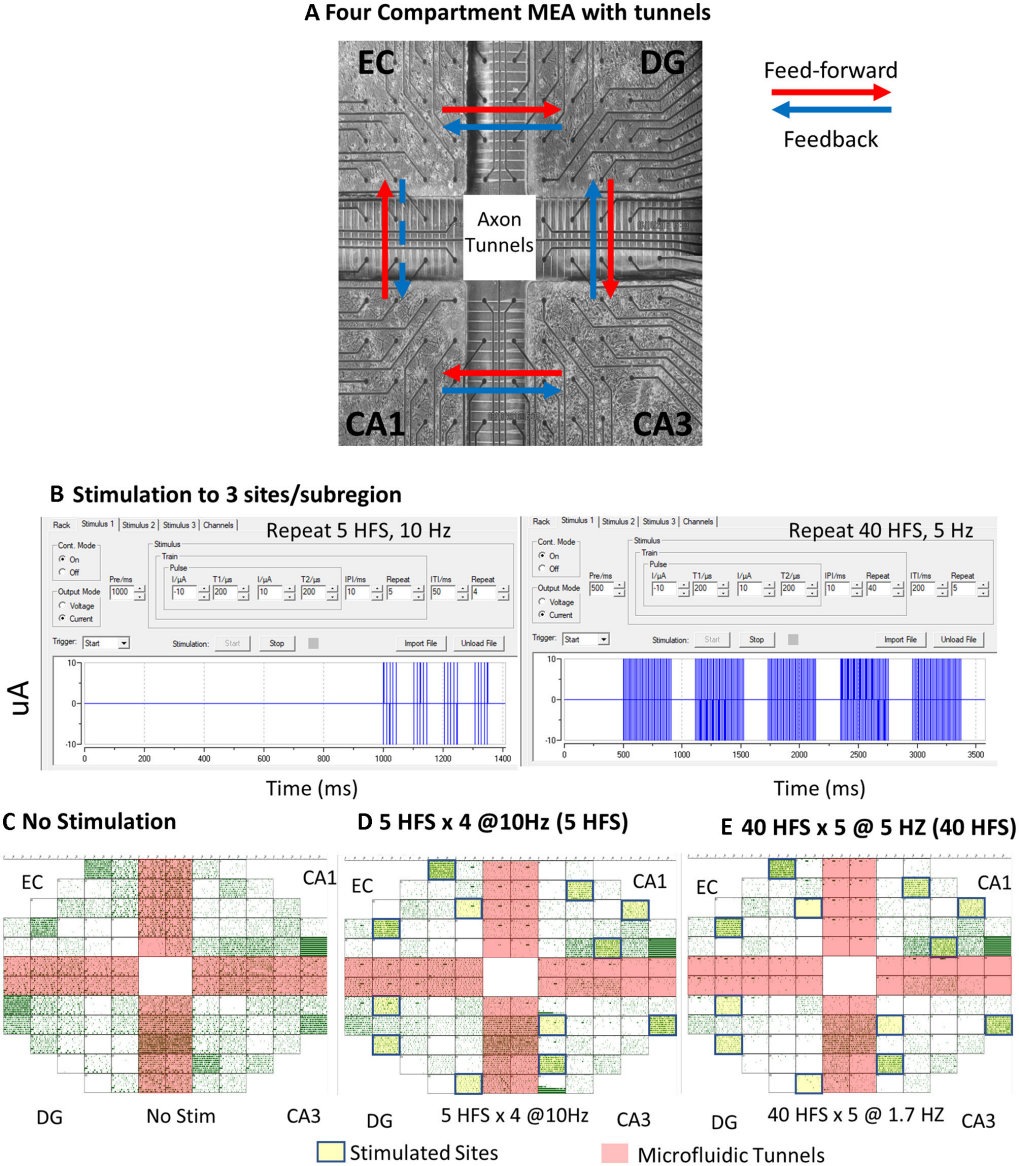
In vitro neuronal culture and spontaneous activity recordings under patterned stimulation conditions. **(A)** Neurons after 3 weeks in culture on a multi-electrode array (MEA). Black circles are 30-μm-diameter electrodes, spaced at 200 μm. Large cross is 1-mm-high PDMS device with microfluidic tunnels 3 × 5 × 400 μm for inter-regional axonal communication. Red arrows indicate the feed-forward direction in this loop, and blue arrows are feedback. EC-CA1 is denoted with a dashed blue line and marked as feedback for internal consistency, but it is more likely an excitatory pathway from EC to CA1. **(B)** MC_Rack programming of the 5-repeat high-frequency stimulation (HFS) stimulation series and 40 HFS stimulation series. Array ECDGCA3CA1 19908 150729 150823 d25 (FID 6). **(C)** Spontaneous recording raster plot generated in MC_Rack of a neuronal culture without stimulation. Each green dot represents a spike. Salmon cross-shading are axon tunnel regions. **(D)** Raster plot of spontaneous spikes after four trains of five biphasic high-frequency pulses spaced at 10 Hz (5 HFS). Yellow boxes were sites of stimulation. **(E)** Spontaneous recording after 5 trains of 40 biphasic high-frequency pulses spaced at 5 Hz (40 HFS).

Here, we gained access to communicating axons between sub-regions, as in Vakilna et al. (2021) by employing a novel four-chamber device with microfluidic tunnels for axonal communication between each chamber where micro dissections of the EC, DG, CA3, and CA1 were cultured (Figure 2A). This 4-chamber device was integrated into a microelectrode array (MEA) in such a way that 5 of the 25 tunnels under each of the 4 partitions separating the chambers were directly aligned over 2 electrodes. The rest of the 120 electrodes were evenly distributed among the 4 chambers. The two electrodes that spanned each of the tunnels allowed the detection of the direction of axonal information propagation, which was used to determine the feed-forward and feedback direction of activity between sub-regions. This successfully enabled the reconstruction of the trisynaptic loop through self-wiring circuits. The first analysis of our four-chamber cultured hippocampal neural networks showed *spontaneous* directional spatiotemporal dynamics in soma and axons within and between the sub-regions of the trisynaptic loop that aided in uncovering the architecture and information transfer strategies of the hippocampus. The underlying distributions of interspike intervals (ISIs), interburst intervals (IBIs), spikes per burst (SPB), burst duration (BD), and intraburst spike rate (IBSR) were determined and were shown to be significantly different between sub-regions. Spontaneous ISIs, IBIs, and SPBs were found to be log–log-distributed, while BDs and IBSRs were log-normally distributed. In the present study, these distributions were used as models for comparison of networks under different stimulation conditions. We also analyzed the functional connections between well electrodes and tunnel electrodes using directional graphs of spike rates that could measure connection strength (Vakilna et al., 2021). Here, we expanded on this study by applying electrical stimulation to probe the plasticity of the circuit and further developed graph analytic metrics for insight into sub-region-specific precise routing strategies.

### Four-chamber *in vitro* hippocampal neuronal network culture

Details were provided previously in Vakilna et al. (2021). Briefly, MEA120 glass multielectrode arrays (MEA) with 120 30-μm-diameter electrodes spaced 0.2 mm apart were used as substrates for the culture of neuronal networks (Multichannel Systems, Reutlingen, Germany; ALA Scientific, Farmingdale, NY, USA). The MEA was divided by a custom polydimethylsiloxane (PDMS) device into four sub-regions each of 9.7 mm^2^ by 1-mm-high wells and 51 microfluidic tunnels 3 μm high × 10 μm wide × 400 μm long spaced 50 μm apart (Figure 2). In total, 5 of the 51 interregional tunnels were monitored with pairs of electrodes to determine the direction of action potentials in single axons. This dedicated 19 electrodes into each sub-regional well. After oxygen plasma treatment of the substrate, the wells were coated with poly-D-lysine for cell adhesion. Hippocampal sub-regions were micro-dissected from postnatal day 4 Sprague Dawley rat pups under anesthesia as approved by the UC Irvine Institutional Animal Care and Use Committee. Brain cells were dissociated and plated at 1,000 cells/mm^2^ for DG (including the hilus), 330 for CA3, 410 for CA1 (including subiculum), and 330 for EC, mimicking the ratio of neural densities *in vivo*: EC-DG 1:3, DG-CA3 3:1, CA3-CA 11:1.25, and CA1-EC 1.25:1 (Braitenberg, 1981). The cells in 10 μL of NbActiv4 medium (Transnetyx BrainBits, Springfield, IL; Brewer et al., 2009b) were plated into the wells sequentially to allow for adhesion before the dish was filled with medium after 30 min in the incubator. The cultures were capped with Teflon membrane covers (ALA Scientific, Farmingdale NY) and incubated for 21–26 days in humidified 5% CO_2_ and 9% O_2_ (Brewer and Cotman, 1989). Half of the medium was changed every 7 days. Activity was recorded 2–5 days after a medium change when the networks reached maturity.

### Multielectrode array recording and patterned stimulation

Spontaneous 5-min recordings and stimulations were produced from the 120-electrode microarray with a Multichannel Systems MEA 120 1100x amplifier (Multichannel Systems, Reutlingen, Germany) at a sampling rate of 25 kHz at 37°C in humidified 5% CO_2_ and 9% O_2_ (custom Airgas USA, Santa Ana, CA). Recordings were initiated several minutes after the removal of networks from the culture incubator, shortly after stable activity was seen in 80% of the tunnels. Arrays with <80% active tunnels or that had poor growth in one of the sub-compartments were rejected for analysis. The Multichannel Systems software MC_Rack was used to capture and initially analyze the recordings and stimulate the culture. Stimuli were biphasic at 200 μs, each with current amplitudes first at −10 μA and then at +10 μA. Motivated by stimulation patterns for induction of LTP in hippocampal slices (Zhu et al., 2015), two kinds of stimuli were delivered at three sites of spontaneous activity. They were patterned within a train containing high-frequency pulses spaced at 10 ms, repeated 5 times (100 Hz), and trains spaced 50 ms apart with four train repeats (termed **5 HFS**) (Figure 2). The second pattern was also 100 Hz pulses spaced at 10 ms, but repeated 40 times, and trains spaced 200 ms apart with 5 train repeats (termed **40 HFS**). Stimuli were applied at three sites in each sub-region in the array with the next train starting as the previous train ended. Stimulation sites were chosen by observing electrodes with sufficient spontaneous activity before stimulation at least two electrodes apart. Spontaneous recordings 67 ± 20 min (SD) after stimulation are reported from 9 separate cultures (9 arrays) listed in Supplementary Table 1; we collected the effects of stimulation for 6 of these. Clockwise direction of plating: (CW) was EC-DG-CA3-CA1. Counterclockwise (CCW) was CA1-CA3-DG-EC to control for plating order bias. None was detected.

### Spike detection, sorting, and axonal propagation direction pipeline

We made automation improvements to our axonal spike directionality algorithm (Vakilna et al., 2021). Raw tunnel data sampled at 25 kHz were filtered through Wave_Clus (Chaure et al., 2018), which uses a second-order elliptic bandpass filter at 300 Hz to 10,000 Hz for spike detection and a fourth-order elliptic bandpass filter at 300 Hz to 3,000 Hz for clustering. Spike detection and clustering were computed above two different thresholds: 5 to 50 S.D. noise and 50.1 to 500 S.D. to ensure that large spikes in the axons were accurately counted and clustered. Through Wave_Clus, the negative peak of spikes was detected with a minimum of 1 ms before the peak and 2 ms after the peak. A refractory period of 1.5 ms was specified. Spike shapes differing by at most three standard deviations from the mean spike shape were included in a single cluster. Clustering was primarily used to discard complex spikes from two or more axons in a microtunnel causing overlapping spike waveforms. All spikes collected from a single electrode in high and low clustering were consolidated into a single cluster. As previous analyses of these tunnel devices showed that ~63% of tunnels only had one axon (Narula et al., 2017), we could identify single axons by their uniform conduction velocities, that is, by spike timing delays, which distinguished multiple axons per tunnel. We empirically checked waveforms after “clustering” through timing delays to ensure singular waveforms. Axonal conduction time delay was used to generate a **normalized matching indexing (NMI)**. With two electrodes spanning the same distance in each tunnel, a timing comparison was made, and an NMI algorithm was computed for every tunnel.


$$\begin{array}{l} NMI=\frac{\#\;paired\;spikes}{\max\left(total\;\#spikes\;per\;spike\;train\right)} \end{array}$$

Tunnels were considered valid for NMI > 0.2 (20% of spikes matched between two clusters), which was sufficient for eliminating spurious spike pair correlations during high spike rates. Paired spikes were identified as spikes with a delay between 0.2 and 1 ms over the 0.2 mm distance, corresponding to the physiological bounds of axonal propagation velocities of hippocampal action potentials (0.4–2 mm/s) (Colombe and Ulinski, 1999). A histogram of conduction times was created with empirically determined thresholding and peak prominence values using the MATLAB findpeaks function. The threshold used was 1.9 standard deviations, and the peak prominence parameter was at least 12% of the highest bin. Peaks in the histograms were identified as belonging to different axons ±0.16 ms, validated with spike shapes. Delay times up to 0.12 ms were considered if there was a peak at sufficiently fast conduction times. Positive delay times indicate feed-forward axons while negative conduction times indicate feedback axons. Example histograms of four types of measured spike timing delays for a given tunnel, and the associated waveforms are provided in Supplementary Figure 1. This is the basis for our determination of the feed-forward and feedback directionality of axonal communication. In contrast to the tunnels, in the wells, raw data were filtered through Wave_Clus and spikes were detected at ±5 S.D. noise. In the wells, neuronal somata density and electrode placement were sparse enough to detect a single neuron in >90% of cases, so spike clustering was not needed.

### Measured spike dynamics and probability distributions

As in Vakilna et al. (2021), the distribution of inter-spike intervals (ISI), inter-burst intervals (IBI), and spikes per burst (SPB) follow log–log distributions and were visualized as normalized complementary cumulative probability distributions (CCDs) with logarithmically spaced bins (Newman, 2005). A log-transformed linear model was used to fit the CCD after log transformation.


$$\begin{array}{l} \log_{10}\left(P\right)=\alpha \times \log_{10}\left(t\right)+c \end{array}$$

where P is the cumulative probability, α the slope, t is the interspike interval in ms, and c is the intercept. The best fits were found by performing a grid search to find the local maximum (highest R^2^) with time limits varied up to 50% with a step size of 5%. For ISI, a single fit was found for all CCDs considering probabilities from 1 to 0.1 and intervals from 0.01 to 1.0 s except EC-DG feed-forward axons which were fitted for intervals between 0.1 to 0.2 s to account for non-linearities in the distribution. For inter-burst intervals, two linear fits were calculated for all CCDs piecewise to account for the “up states” and “down states,” which refer to fast and slow bursting, respectively (Vakilna et al., 2021). The minimum time for the up states was used as the maximum time for the down states. The distributions of intra-burst spike rates and burst durations better followed a log-normal distribution and were visualized with a probability distribution (Vakilna et al., 2021). The median was calculated by fitting a normal distribution to the probability distribution in natural log space. The mean in natural log space (m) was extracted from the fit. A one-way ANOVA was calculated from the fits.


$$\begin{array}{l} Median\;of\;lognormal\;distribution=e^{m} \end{array}$$

### Edge plot graph analysis

In graph analysis, edges are the connections between nodes (neurons and tunnel axons). Edge detection was used to correlate firing rates between well and tunnel electrodes. NMI was designed to be very exact and discriminatory for spike identification and required robust signals from both electrodes in a tunnel. However, examining raster plots, some channels exhibited signs of lower axonal coupling to the electrode. Therefore, the more active electrode was used as the basis for edge detection. The average firing rate for each 100 ms bin was calculated from all combinations of each axon in a tunnel and each adjacent well electrode with a 10-s-wide sliding window using 100-ms steps. If the window contained at least four bins with a non-zero firing rate in a combination of well and tunnel, a linear regression correlating the wells and tunnels was calculated. This was repeated for all possible connections. For plotting, all linear regressions with a positive slope >0.1 and an R^2^ >0.2 were used. A slope <1 indicated a decreased firing rate through that connection and a slope >1 indicated an increase in firing rate. The reliability of this connection was described through the R^2^ values (Pearson linear regression). These values were averaged over all the time windows for each array to derive weights and graphically depicted to generalize the active connections in the network. MATLAB graph analysis functions were employed to calculate the number of edges (connections from a well node to a tunnel axon node), degrees (number of edges per node), and centrality (degrees weighted for slope or R^2^) of each node for feed-forward and feedback connections for well-to-tunnel edges.

### Statistics

Data were analyzed with custom MATLAB 2020a scripts. Slopes were compared for a statistically significant difference (α < 0.05) using analysis of covariance (ANCOVA) and Tukey's honest significant difference (HSD) test. The significance within a stimulation set between sub-regions and between stimulation sets of the same sub-region was analyzed by analysis of variance (ANOVA) and Tukey's HSD test with the null hypothesis rejected for *p* < 0.05. Data were analyzed for nine separately plated networks, six of which were subjected to patterned stimulation. Details are summarized in Supplementary Table 1.

## Results

### Different sub-regional and axonal spiking and bursting dynamics in response to patterned electrical stimulation in the self-wired hippocampus

In order to reveal the architecture and the dynamics that govern hippocampal information processing, we designed a four-compartment device with microfluidic tunnels that promoted axonal self-wiring between sub-regions and prevented dendritic infiltration (Figure 2A). As discussed in the introduction, traditional methods of studying the encoding and decoding of hippocampal information rely on interpreting behavior in conjunction with electrical recording. However, these studies often pay asymmetric attention to one part of the hippocampus over another and do not study the differences in dynamics of information encoding in each sub-region of the hippocampus or in control of the axonal information that gets transmitted from one region to the next. This is due to the difficulty of accessing all regions of the hippocampus simultaneously and the impossibility of isolation of recordings from single axons during *in vivo* recordings. The four-compartment design allows for direct access to the computations both within each sub-region and the axons between them, which makes high-resolution decoding of dynamics easier to interpret. Each compartment contains a sub-region of the tri-synaptic loop (EC, DG, CA3, and CA1) and tunnels between each sub-region for their communication. Alignment over a multi-electrode array allowed for recording of spontaneous activity and two electrodes spanning tunnels allowed for monitoring of axonal activity in 5 of the 51 tunnels between sub-regions with a majority containing only 1 or 2 axons. Nine arrays were included for spontaneous unstimulated activity. In total, 6 of those arrays were electrically stimulated with 5 pulses at 100 Hz at theta frequency repeats (5 HFS), followed by measures of spontaneous activity, then 40 pulses at the same 100 Hz at theta frequency (40 HFS), which comprise short and long high-frequency stimulation (Figure 2B). The 40 HFS was again followed by a 5-min recording of spontaneous activity for a total of three spontaneous recordings (pre-stimulation, post 5 HFS, and post 40 HFS).

We expected these reconstructed networks to operate around a critical point between an ordered and a random system, which optimizes information processing functions (Beggs and Timme, 2012). Critical networks produce avalanches of activity that follow power law distributions. In order to maintain this balance around the critical point, a closed loop network could balance each sub-region or exhibit sub-regionally specific network dynamics responses to optimize information transmission of the whole. The patterned stimulation was designed to probe short-term plasticity to determine whether the network dynamics shifted in unison or specific sub-regions exhibited unique responses. Figures 2C–E shows raster plots from before and after stimulation. Decreased network spiking was seen in EC and DG, CA3 and CA1 sub-regions as well as in the EC-DG, CA3-CA1, and CA1-EC axons. Detailed dynamics analysis of multiple array networks was needed to be confident of significant differences in interspike intervals (ISI), interburst intervals (IBI), spikes per burst (SPB), burst duration (BD), intraburst spike rate (IBSR), and connectivity graphs for critical network balancing across sub-regions. At this subjective level for one array, network spiking appeared to decrease as a result of stimulation (models in Figure 1C or Figure 1D, but not Figure 1B).

### Spike dynamics revealed that stimulation lengthens interspike intervals in sub-regions and communicating axons with greater net feedback signaling

**Figure 3:**
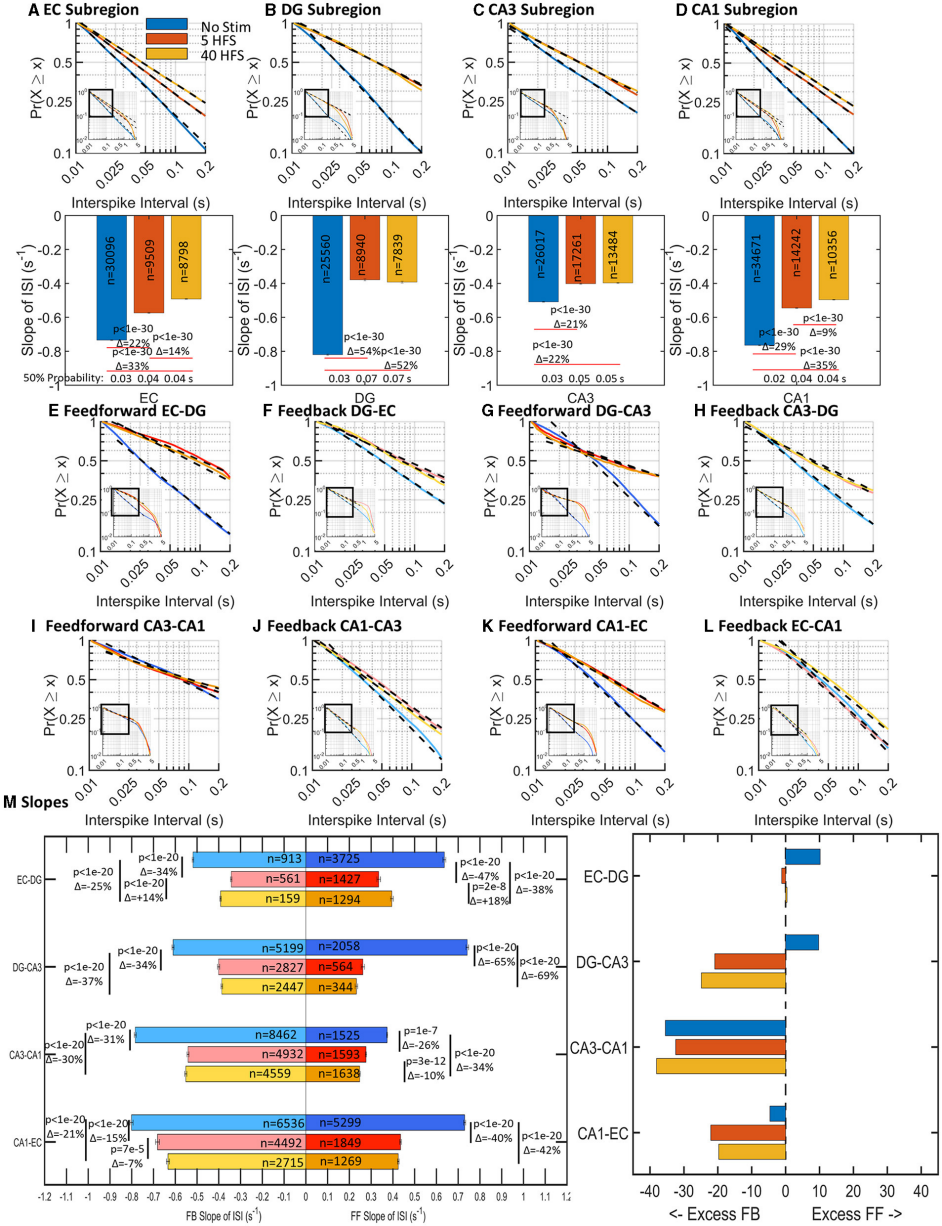
Stimulation decreased slopes of log-log interspike interval (ISI) distributions (slowed spike rates) in all sub-regions and communicating axons (*n* = 9 unstimulated arrays, 6 arrays stimulated at each of 5 HFS, and 40 HFS). Insets show linear fits over nearly two orders of magnitude. **(A–D)** ISI slopes decreased after stimulation (decrease in spike rate) in each sub-regional well. Embedded numbers are spikes per array. Delta percent change in slope and ANOVA indicated. **(E–L)** Adjacent displays of feed-forward and feedback axons in tunnels to compare net differences. Most but not all axon ISI slopes declined with stimulation. **(M)** Differences in feed-forward and feedback directional slope changes in each set of communicating axons. The number of ISIs detected, n, is shown as well as the Delta percent change in slope and ANOVA. (Right) Percent differences between feed-forward and feedback axons in the same tunnels show an overall increase toward feedback spiking.

Neuronal spiking in communicating axons relays information from one sub-region of the hippocampus to another, with feed-forward likely being excitatory and feedback likely being inhibitory. Some axons in CA3-DG tunnels stained for the enzyme for the GABA synthesis enzyme, glutamic acid decarboxylase (GAD), indicating precedent for inhibitory axon transmission (Brewer et al., 2013). We define feed-forward as EC>DG>CA3>CA1, back to EC in the trisynaptic loop. EC-CA1 is evaluated as feedback for internal consistency, but it is more likely an excitatory pathway from EC to CA1 (Steward, 1976). How spikes are spatiotemporally organized and respond to stimuli may provide insights into the function and behavior of the network. As previously found in Vakilna et al. (2021), spontaneous interspike intervals (ISI) followed a log-log distribution and varied sub-regionally and interregionally. Both before and after applied patterned stimulation, complementary cumulative distributions of ISIs followed a log–log relationship in individual arrays and for the cumulation of spikes from nine unstimulated and six stimulated arrays (Figure 3). The probability (P) of a spike firing at time (t) after a previous spike followed a linear model with slope m and can be described by the formula P = t^m^. Shorter times correspond to faster firing rates so that more spikes with short ISIs will produce a graph with a steeper slope. Longer ISIs have a slower firing rate with shallower slopes. With stimulation, subsequent spontaneous activity 5 min later will either (a) be unchanged because the network forgot the stimulus and reverts to a resting state or (b) the overall spike rate of the network will adjust to the additional information and increase the overall spike rate (steeper ISI slope) or (c) decrease spike rates because the stimulus-induced selective routing in the network (shallower ISI slope). Selective routing forces a reduction in possible routes for information to pass through and “sharpens” the routing of the network.

Our unique device architecture (Figure 2) allows monitoring of network activity in isolated axons that communicate through the 51 microfluidic tunnels between each sub-region. Five of these tunnels are monitored as their resident axons pass over two microelectrodes in each of these tunnels. The dual electrodes enable the determination of the direction of spike propagation by spike timing differences (Vakilna et al., 2021). Thus, Figures 3E, F shows the spike timing in axons carrying information in the feed-forward direction from EC to DG (Figure 3E, EC-DG) and feedback direction from DG to EC (Figure 3F, DG-EC). These and all other axons (Figures 3E–L) showed decreased slopes with stimulation. Further comparison of Figures 3E, F indicates a greater decline in feed-forward EC-DG slope with stimulation than the smaller change with feedback in DG-EC. To gain insight into the relative directional changes in network communication between sub-regions with stimulation, this relative excitation–inhibition balance is more readily seen in Figure 3M. The excess feed-forward activity from EC-DG and DG-CA3 in the unstimulated network (blue bars) was balanced by excess feedback activity in the CA3-CA1 and CA1-EC axons. After stimulation, this balance was shifted to excess feedback in DG-CA3 and increased CA1-EC feedback (which we will show later is more likely EC-CA1 feed-forward). By sub-region, distinct slopes were observed in most of the sub-regions in response to each of the stimulation conditions and feed-forward activity proportionally followed well sub-region activity (Supplementary Figure 2). Overall, the network retained balanced control by adjusting feed-forward (FF) and feedback (FB) axonal directionality after stimulation accomplished by decreased spiking (lower n) and decreased slopes of the log-log ISIs (longer times between spikes). These results are consistent with Figure 1C, a decrease in spike rates because the stimulus-induced excess feedback over feed-forward activity. We next determined how the grouping of spikes into bursts might contribute to the specificity of the network communication.

### Burst rates decrease after stimulation and uniquely balance dynamics sub-regionally and interregionally

**Figure 4:**
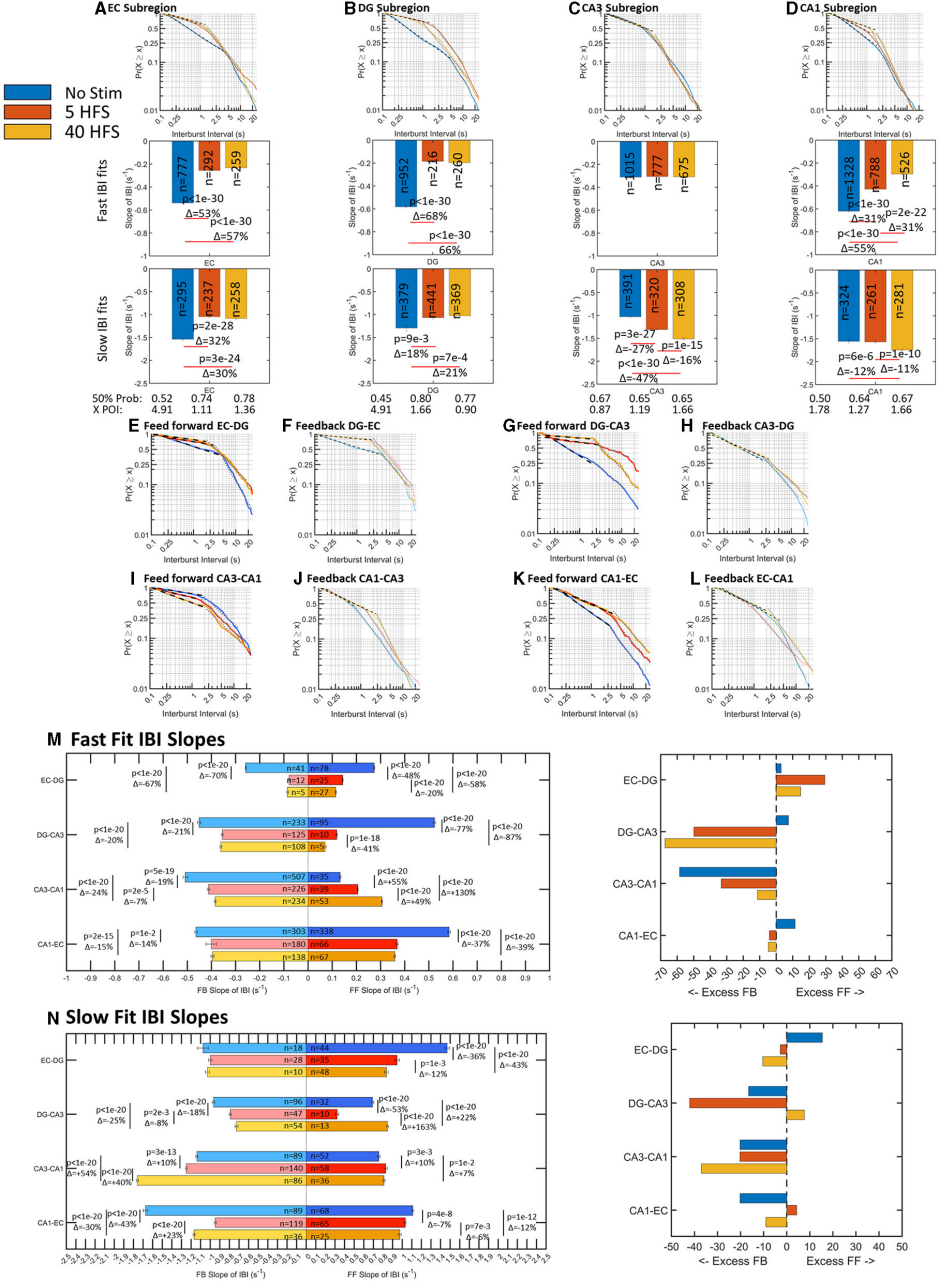
Burst rates in wells and tunnels decrease after stimulation. Interburst intervals (IBI) follow log–log distributions with two linear fits for all stimulations that vary by sub-region with stimulation (n arrays = 9 unstimulated, 6 at 5 HFS, and 6 at 40 HFS). A decrease in slope is a decrease in burst rate. In general, networks shift toward slower bursting after stimulation, except CA3. **(A–D)** Sub-regional fast IBI slopes decrease >40% after stimulation in all regions except no change in CA3. For slow IBI, EC and DG slopes decreased; CA3 slope increased, and CA1 remained similar. In EC, DG, and CA1, the 50%-line increased after stimulation indicating slower bursts detected. CA3 50% remains similar. The point of intersection between fast and slow slopes decreased from 5s to 1s after stimulation in EC and DG, while CA3 was not changed. **(E–L)** Feedforward and feedback slopes are placed adjacent to each other to compare the net changes in the distribution before and after stimulation. Generally, the slopes decreased and the point of intersection between the fast and slow regimes moved toward faster spike rates. This means the slow bursting regime included faster bursting intervals. **(M)** Comparisons of feed-forward to feedback slopes for fast bursts favored feedback, but CA3-CA1 feed-forward increased with stimulation. Fractional changes between feed-forward and feedback trended toward excess feedback. **(N)** Comparisons of feed-forward to feedback slopes for slow bursts. Again, fractional changes between feed-forward and feedback trended toward excess feedback.

Spikes are organized into bursts or packets of information for both local and global computation and routing (Graham and Rockmore, 2011; Graham, 2014; Luczak et al., 2015). Bursts of spikes are needed to raise the somal calcium (Jimbo et al., 1993) that is readily recorded in localized regions *in vivo*. Hippocampal architecture is organized so that each sub-region is responsible for a different set of computations that give rise to learning and memory (Kesner and Rolls, 2015). Knowing the spatiotemporal bursting dynamics in sub-regions and axons allows for insight into how each sub-region routes these information packets to be processed downstream. Previously, we found spontaneous interburst intervals (IBI) followed a two-part log–log distribution suggesting faster up and slower down states that vary sub-regionally and interregionally (Vakilna et al., 2021). Like ISI, when there is a decrease in slope the frequency of bursting decreases. After we applied 5 HFS and 40 HFS stimulation, fast bursting generally decreased and slow bursting increased (Figure 4). The bursts were plotted over a timescale of two orders of magnitude encapsulating two orders of probability vs. times that a bursting event will occur. In the sub-regions, Figures 4A–D shows slopes in the fast-bursting domain of 0.1–1s decreased 31–68% in EC, DG, and CA1, while CA3 remained unchanged with stimulation. In the slower bursting domain of approximately 2–20 s, early network burst slopes in EC and DG decreased 18–32%, while late network burst slopes in CA3 and CA1 increased 12–47% with stimulation. Stimulation also affected the burst timing of communicating axons between sub-regions in ways specific to the direction of axon propagation (Figures 4E–N).

We next focused on the fast bursts as they represent the majority of events and present the slow fit changes for completeness. The changes in slopes with direction were most easily viewed in Figures 4M, N. Before stimulation (blue bars), the network balanced excess feed-forward fast bursting in EC-DG, EC-CA3, and EC-CA1 with feedback bursting in CA3-CA1. The difference between the slopes for the fast and slow bursts shows in which direction the speed of bursting was dominant. Before stimulation, comparison of feed-forward to feedback for EC-DG, feed-forward slopes of the fast and slow distributions exceeded the feedback slopes by 3.0 and 15%. After stimulation, feed-forward fast slopes declined by 48 to 58% while feedback slopes declined by 67–70%. Thus, the feedforward–feedback balance in EC-DG axons shifted with stimulation to greater feed-forward transmission. In contrast, DG-CA3 axons shifted from 7.4% excess feed-forward over feedback before stimulation to a much greater effect of stimulation on feed-forward axons (77–87% shallower slope) than feedback axons (21–20% shallower slope) for a change in the net balance toward 50 and 67% excess feedback transmission of bursts in DG-CA3 after stimulation, respectively. For the next set of axons between CA3 and CA1, feedback burst signaling exceeded feed-forward by 59%. Stimulation caused an increase in slopes of the feed-forward axons of 55 and 130%, while feedback slopes decreased by 19 and 24% toward a more balanced feed-forward and feedback bursting. For fast CA1 to EC bursts, feed-forward exceeded feedback bursts by 11% before stimulation. With stimulation, the 37 and 39% lesser feed-forward slopes and 14–15% lesser feedback slopes brought CA1-EC axons into closer feed-forward–feedback balance. Fractional differences between fast and slow bursting (Supplementary Figure 3) show that feed-forward axons and wells tend to shift from fast bursting before stimulation to slow bursting after stimulation except for the feed-forward CA3-CA1. Taken together, these log–log relationships of interburst intervals indicate a parceled form to balance bursting among the inter-regional axons of the network with a shift from unstimulated control of fast feed-forward bursting by CA3-CA1 feedback, to stimulation-induced decreases in this CA3-CA1 feedback, and increases in DG-CA3 feedback in response to net increases in EC-DG bursting. For the slower burst type, the frequency of feedback bursting greatly exceeded feed-forward bursting. The emphasis on feedback bursts for incoming and outgoing CA3 axons may be to provide brakes on the recurrent collateral excitation in the CA3 (Bains et al., 1999). In the context of the packet metaphor, a decrease in faster bursting rates could be an indication that the network is now more selective for what information it sends into the next sub-region for computation consistent with option C (Figure 1), and stimulus-induced selective routing in the network, a route sharpening.

### Spikes per burst decreased in EC-DG axons and soma after stimulation for smaller packet sizes for routing information

**Figure 5:**
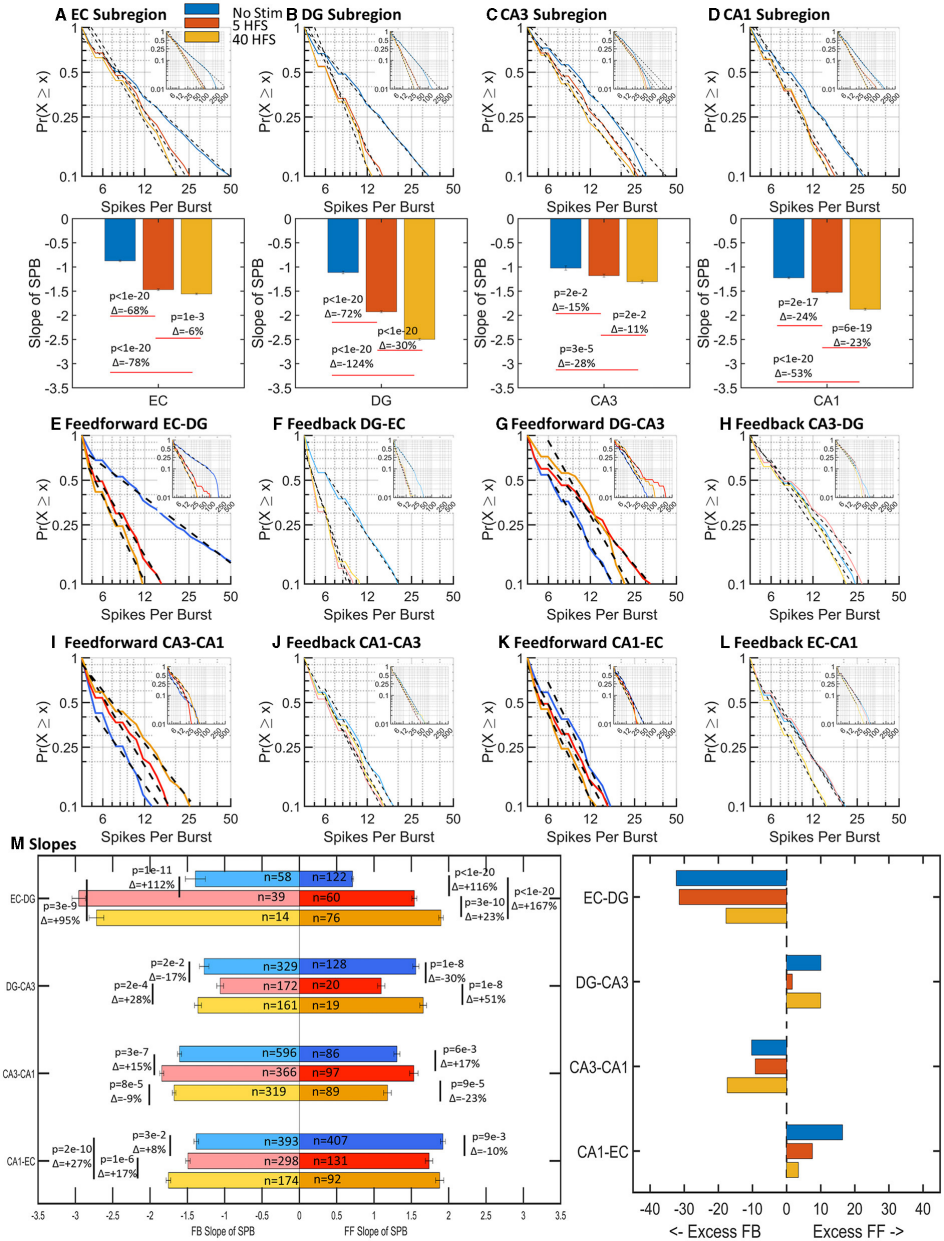
Spikes per burst (SPB) followed log–log distributions for all stimulations and vary sub-regionally and interregionally with stimulation (*n* = 9,6,6 arrays). In general, there was an increase in slope with stimulations. An increase in slope is a decrease in the number of spikes per burst. **(A–D)** Sub-region well spikes per burst slopes increased with stimulation in all cases, indicating a decrease in the number of spikes per burst. **(E–L)** Feed-forward and feedback spikes per burst slopes are adjacent for comparison of net differences. **(M)** Feed-forward and feedback axonal spikes per burst increased in EC-DG axons after stimulation. Fractional differences in slope trend toward excess feed-forward in the DG-CA3 and CA1-EC tunnels while the EC-DG and CA3-CA1 tunnels have excess feedback. Excess refers to the magnitude of slope in one direction. In the case of spikes per burst, an excess in slope magnitude would mean fewer spikes per burst in that direction.

If a burst of spikes is analogous to a packet of information, then the number of spikes within a burst is analogous to the amount of information contained within the packet. We expected brain networks would balance their dynamics to optimize cost (energy) and efficacy. As shown in Figure 5, the number of spikes within a burst was log–log distributed and ranged between 4 and 50 spikes for 90% of the bursts. With stimulation, the 15–124% increase in slopes in sub-regions (Figures 5A–D) indicated a decrease in the number of spikes per burst. After stimulation, 90% of sub-region bursts contained 12–25 spikes, which was half to a quarter of spikes before stimulation.

Before stimulation, feed-forward axons in the EC-DG ranged from 4 to over 50 spikes per burst (Figure 5E). Other feed-forward axon bursts contained fewer than 20 spikes (Figures 5G, I, K). After stimulation, EC-DG spikes per burst decreased greatly in EC-DG and slightly increased in all other axons between sub-regions (Figures 5E–L). The net balance in the network (Figure 5M) showed large increases in slopes of 95–167% for both feed-forward and feedback in EC-DG greatly exceeding the small variations in the other axons between sub-regions. Overall, stimulation increased the spikes per burst most dramatically in EC-DG feedback axons and their target DG neurons, which may promote greater downstream signaling into the network, a refinement to the idea of route sharpening (Figure 1C).

### Bursts as packets of information deliver information faster in a shorter period in response to stimulation

The brain has been compared to the internet network architecture because of its capacity to route information from one node to another across robust distributed networks. Communication protocols that facilitate information exchange on the internet use packet-switched networks (PSNs), which chop up a message into smaller chunks that are recombined at the destination. As described by Graham and Rockmore (2011), collections of neurons can act analogously to nodes in internet network architectures in that there is a neural hierarchy with specific cells performing certain functions, a multitude of encoding mechanisms allow for simultaneous applications to run on the same network and that its distributed architecture allows high-bandwidth computations to occur without large, dedicated memory allocations. Neural data packets could then be thought of as spikes or bursts. We have studied how the distribution of these packets is modulated with stimulation, but how these packets are structured may give insight into how the network modulates its routing capabilities in response to stimulation.

**Figure 6:**
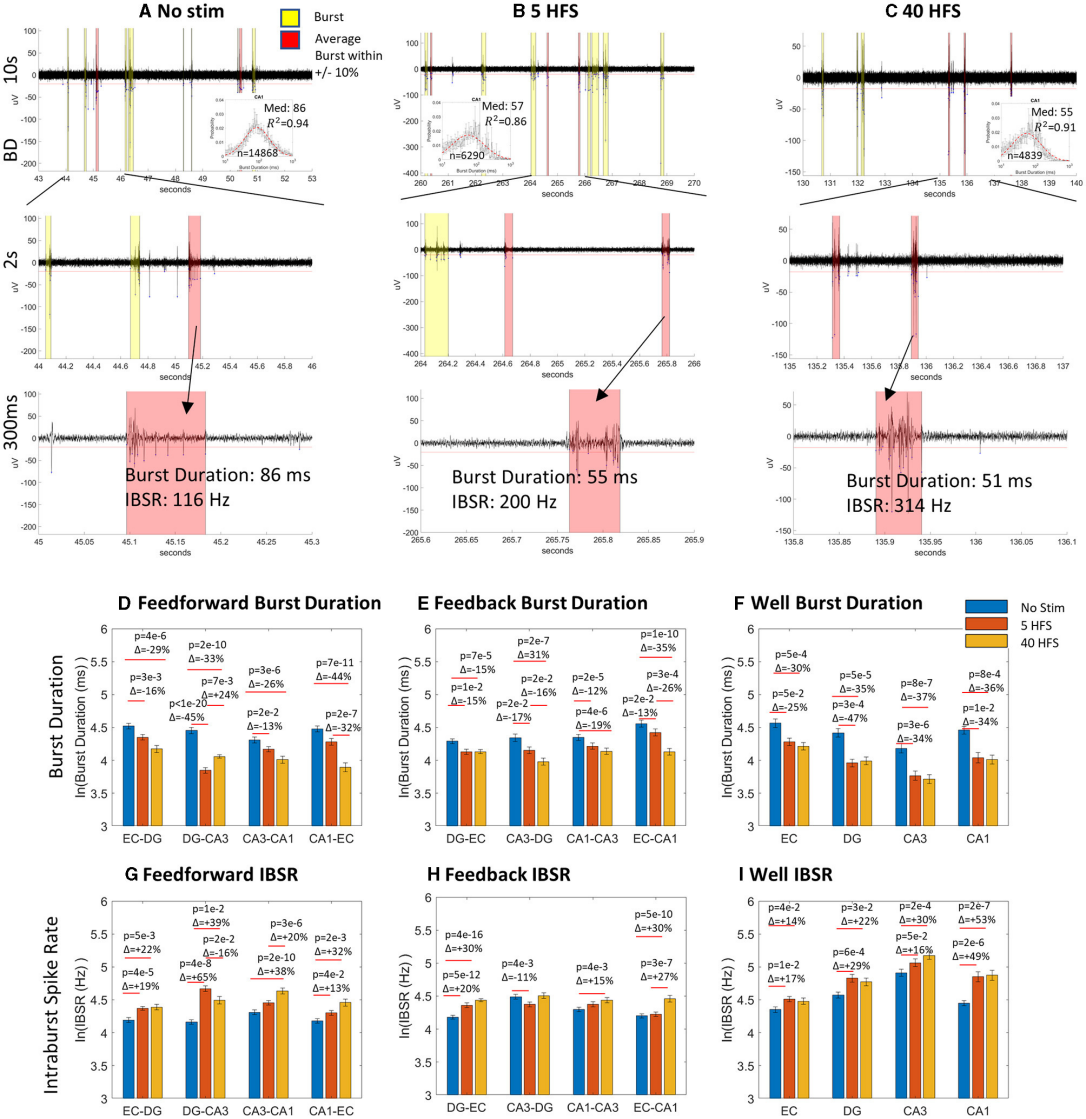
Stimulation evoked decreased burst duration and increased intraburst spike rate. **(A–C)** Raw CA1 well data at 10 s, 2 s, and 300 ms timescales at a point of average spiking activity for no stimulation, 5 HFS and 40 HFS, respectively. Inset semi-log histograms show decreased burst duration. Bursts close to the average length are shown in salmon highlights, others in yellow. **(D–I)** Burst duration decreased from no stimulation in seven of eight feed-forward axons, seven of eight feedback axons, and all well channels for no stimulation. Intraburst spike rates increased in six of eight feed-forward axons, five of eight feedback axons, and all sub-region well channels from no stimulation. Statistics were calculated from Gaussian fits.

Figures 6A–C shows the raw data in well electrodes for how the same channel's median spike rate ±10% is modulated with stimulation. With each stimulation progression from no stimulation, the bursts got shorter, and the spike rate increased. There are not necessarily more spikes in a burst that increase the intraburst spike rate, as shown in Figure 4, but they are condensed into a tighter time window. This gives the impression that the network balanced new stimulus information with smaller packets of less information, but the information was transmitted at a faster rate when a packet arrived. This suggests synapse strength was potentiated by increased synaptic calcium concentrations (Emptage et al., 1999), as bursts raise intracellular calcium (Jimbo et al., 1993). Figures 6E–J supports this assessment across almost all tunnel and well electrodes. Compared to before stimulation, burst duration after stimulation decreased in all feed-forward axons (Figure 6E) and feedback axons (Figure 6F). Full semi-log histograms supporting these averages are shown in Supplementary Figure 4. For the sub-regional channels (Figure 6G), all burst durations decreased essentially equally from the no-stimulation condition. For intraburst spike rates (Figures 6H–J) feed-forward rates all increased from no stimulation (Figure 6H). These increases were partly balanced by increased feedback intraburst spike rates (Figure 6I) from no stimulation for both stimulations in DG-EC, CA1-CA3 HFS 40, and EC-CA1 HFS 40. Feedback intraburst spike rates decreased for CA3-DG HFS 5. In the well sub-regions (Figure 6J), all intraburst spike rates increased from no stimulation essentially equally, following the same trend as in the burst duration. The semi-log histograms of intraburst spike rates supporting these medians are provided in Supplementary Figure 5. Overall, we saw shorter packets of information being delivered at a faster spike rate, a newly revealed way to sharpen the routing of information, consistent with Figure 1C.

### Graphic analysis of functional connectivity: small decreases after stimulation with a balanced ratio of feed-forward vs. feedback connections

In our hippocampal cultures, we have the ability to sample 19 out of thousands of neurons in each sub-region and monitor approximately 10% of the axonal tunnels. Therefore, it is highly unlikely that a neuron we are recording from has a direct axonal connection through one of these tunnels, though a connection through a single synapse would be more likely. To search for functional connections of activity between the well neurons and the tunnel axons, we looked for repeated incidents of coordinated spiking activity over short time scales. We created linear regressions of spike rates between the sub-region well and tunnel axons. Supplementary Figure 6 provides examples of high- and low-windowed correlations. When computed for all combinations between sub-region neurons and tunnel axons, we graphed functional connectivity on a fixed electrode topology for each MEA network with each spike rate correlation represented as an averaged weighted directional edge, essentially an input–output relationship. From these correlations, we derived slopes that indicated one of three conditions. (1) Slope = 1 when a neuron directly connected to the measured axon with matched spike rates; (2) slope >1 when the excitatory source neuron contributed to higher target axonal spiking; or slope <1 when the inhibitory source neuron decreased the spiking of the measured target axon.

**Figure 7:**
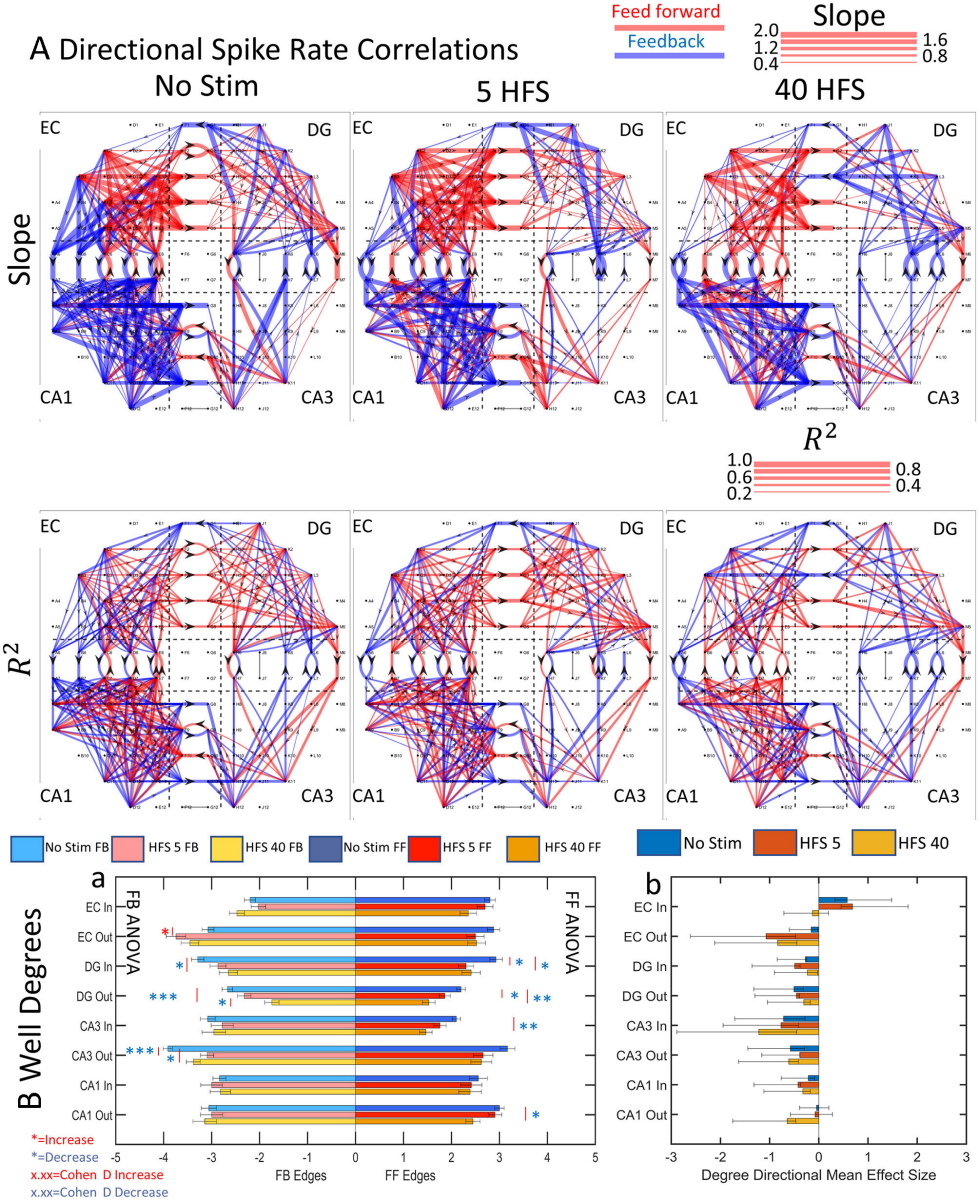
Functional connectivity routing from directional spike rate correlations decreases after the application of patterned stimulation. **(A)** Directional graph exemplifies a decrease in edges with each stimulation from one array (FID 3). The scale indicates the slopes as line widths for each given direction. **(B**a**)** The average edges per node in each sub-region decreased after stimulation in 9 of 16 sub-region directions. *n* = 9 unstimulated and 6 stimulated arrays. ANOVA indicates increase (red *p < 0.05; **p < 0.001; ***p < 0.0001) or decrease (blue *). **(B**b**)** A mean effect size and 95% confidence intervals were calculated for each sub-regional direction. All sub-regional directions showed excess feedback degrees, suggesting more active functional connections between well->tunnel and tunnel->well channels, in all cases except EC-In and CA1-Out. After stimulation, these sub-regional directions changed from excess feed-forward degrees in the EC-In to balanced feed-forward/feedback degrees in the HFS 40 stimulation. The change in the CA1-Out was from balanced feed-forward/feedback degrees to excess feedback after the HFS 40 stimulation. This suggests different routing strategies for balancing the network activity under different stimulation conditions.

These relationships enabled measures of reliability and connection strength or weight. The reliability was obtained from the reproducibility of the data over multiple time windows fit a linear model with a derived Pearson's R^2^ measure of fit. In our graph analysis, each electrode represents a node. The degree of a node is the number of edges feeding into the node or out of the node. Figure 7A shows slope, connection weights, and reliability for directionally separated feed-forward or feedback edges. Clearly, the network segregated feed-forward and feedback connections differently in different sub-regions and the reliability (reproducibility, R^2^) of the connections varied by sub-region as well.

To compare the sub-regional feed-forward and feedback activity before and after stimulation, average edges per node per array for each sub-region were computed from nine unstimulated and six stimulated arrays (Figure 7B). We separated the incoming from outgoing edges at each node based on the directionality of the tunnel axons to arrive at a well degree metric. From the prevalence of red (or blue) asterisks signaling a significant increase (or decrease) in well degrees after stimulation, the average number of degrees decreased for feed-forward connections in 6 of 8 groups (DG in 5 HFS, DG in 40 HFS, DG out 5 HFS, DG out 40 HFS, CA3 in 40 HFS, and CA1 out 40 HFS) and feedback decreased in 5 of 8 groups (DG in 40 HFS, DG out 5 HFS, DG out 40 HFS, CA3 out 5 HFS, and CA3 out 40 HFS) while only EC-out feedback showed an increase (Figure 7Ba).

The *p*-values are insufficient for quantifying the magnitude of the effect on network dynamics (Sullivan and Feinn, 2012). Significant changes in the ratio of feed-forward and feedback connections are more meaningful if Cohen's *d* of mean effect sizes are calculated for the number of degrees in Figure 7B. For example, for the first sub-region, EC Inward edges without stimulation showed a medium–strong (0.5–0.8) Cohen's *d* of 0.58 for excess feed-forward over feedback that remained unchanged for HFS 5 but was lost with HFS 40. With the exception of CA1 in Figure 7Ba, seven of the other sub-region directions produced an excess of feedback over feed-forward with medium to large (>0.8) effect sizes comparison of feed-forward/feedback balance (likely excitation–inhibition). This shows stimulation to cause a shift to more feedback than feed-forward routes. In 11 of 13 significant differences between feed-forward and feedback well degrees, feedback had more degrees than feed-forward. Overall, these changes suggest that the network directed the routing of activity after stimulation, limiting the number of feed-forward routes over feedback.

### Network graphic response to stimulation: more reliable excitatory and inhibitory pathways with stronger feedback spike rates, a network sharpening

**Figure 8:**
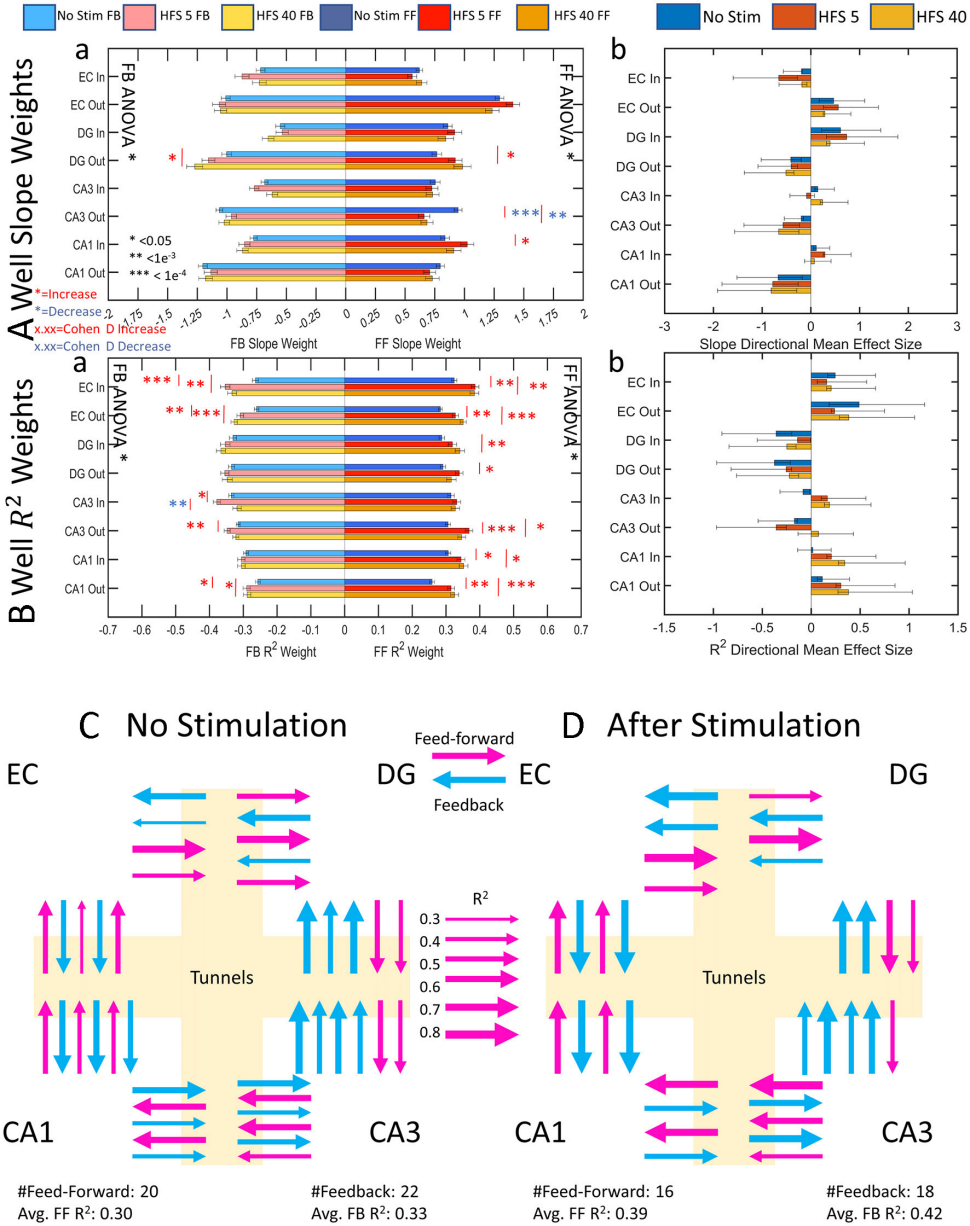
Centrality graph analysis shows increasing feedback specificity and importance, suggesting that stimulation has a “sharpening effect” on network activity where information routing is directed and amplified over feed-forward activity. Mean effect size analyses show in which direction the measured parameter is dominant. Error bars on mean effect size are 95% confidence intervals. **(A)** All significant changes (6) across stimulation conditions decreased except for DG out which increased. Cohen's d showed an increasing effect size toward feedback activity with successive stimulation in nine cases. **(B)** Reliability of feedback stimulation increases in five out of eight cases. Cohen's d effect size measurements increased from no stimulation in 3 cases and favored feedback in 11 out of 13 cases. **(C, D)** Summary cartoon diagrams of directional graphs were created to exemplify decreased edges with increased reliability. **(C)** Before stimulation, feedback is slightly in excess of feed-forward degrees and reliability as in Figure 1A. **(D)** After stimulation, the number of edges decreases but reliability in both directions increases as in Figure 1C. Line weight increases according to the scale of reliability (R^2^).

*In vivo*, feed-forward activity is commonly excitatory, and feedback inhibitory. In our networks, the same control system may be at work to direct the general routing of network activity. Without these braking systems, the network would run away to metabolic exhaustion with continuous feed-forward activity. The values of average functional connectivity strength (number of connected edges) and reliability of a source electrode influencing the spike rate of a target electrode are represented as edge weights in the directional graph (Figure 8Aa). DG out feed-forward and feedback slope weights both increased from no stimulation to HFS 40. CA3 out feed-forward slope weights decreased after both stimulations, and CA1 in feed-forward increased from no stimulation for HFS 5. Otherwise, the other 27 sub-regional directions remained unchanged after stimulation. This indicated that the functional connections maintain a proportional feed-forward-to-feedback balance between the source and target before and after stimulation. The direction of the network remained balanced based on mean effect size analysis between feed-forward and feedback slope weights shows in which direction the network is balanced (Figure 8Ab). EC in, DG out, CA3 out, and CA1 out directions all maintain stronger feedback activity across all stimulation series. EC out and DG in directions maintain stronger feed-forward activity across all stimulation series. CA3 in and CA1 in slope weights produced no consistent feed-forward strength over feedback.

Figure 8B shows the reliability of the functional connectivity. The average increase for feed-forward reliability was 16 ± 2% (mean ± SE) and for feedback reliability 13 ± 2% after stimulation (significance above no change, one-tailed *t*-test, *p* = 10^−8^, *p* = 10^−5^, respectively). An increase in reliability may indicate a preferred pathway, or routing strategy, that the network takes to communicate a consistent input and maintain balance. Feed-forward activity had twelve increases in reliability from no stimulation and feedback had eight increases in reliability from no stimulation (red ^*^, Figure 8Ba). A mean effect size calculation between feed-forward and feedback reliability (Figure 8Bb) showed the network largely maintained its ratios of reliability between feed-forward and feedback save for a few notable differences. Effect size analyses were considered valid in the positive or negative directions when the confidence intervals did not cross zero. Thus, we note that the CA3-in switches from more reliable feedback activity to more reliable feed-forward activity after stimulation with a small effect. CA3 out for HFS 40 was more balanced as opposed to no stimulation and HFS 5, which have feedback reliability dominating. Moreover, CA1-in had balanced reliability before stimulation and became feed-forward dominant with a medium effect size. CA1-out reliability also increased feed-forward dominance from a small effect size to a medium effect size. Overall, the network became more reliably feed-forward after stimulation. We interpret this as a route sharpening.

### The network assigned more importance to feedback activity than feed-forward activity

Centrality in graph analysis is an important measurement of the nodes that make up the network. Higher importance is assigned to nodes that have more degrees of larger weight. In our hippocampal networks, we measured the importance of slope and R^2^ in order to gain insight on how the network assigned importance on routing and controlling the magnitude of excitatory-inhibitory activity and to investigate whether some routes were more important than others. The modulation of slope centrality for maintaining balance while routing information was largely unchanged between stimulations (Supplementary Figure 7Aa). Only 4 of 16 feed-forward significant centrality changed from no stimulation and 2 out of 16 significant changes in feedback centrality, and all decreased from the no stimulation condition. In Supplementary Figure 7Ab the mean effect sizes of centrality, only the EC showed more importance of feed-forward activity over feedback in all three stimulation conditions. This means that the network placed more importance on the EC incoming activity's effect on the network to operate. This is despite in Figure 8A feed-forward and feedback EC in activity are weighted <1, which has an overall dampening effect. EC out showed more feedback slope importance after stimulation, which means the feedback axons from EC to the CA1 were more abundantly active and fired faster with respect to the sub-regional EC activity to EC-DG axons. The rest of the sub-regional directions had a preference for feedback of slope centrality inhibition.

The importance of reliability to induce proportional spike rates in a target node may indicate selectivity of the network in one direction or another and which pathways get more reliably activated for network control. Supplementary Figure 7B depicts mixed effects of stimulation on centrality R^2^ (reliability). Feedback EC-in showed an increase in feedback reliability importance after stimulation at 40 HFS (Supplementary Figure 7Ba). The excess Cohen's *d* for EC-in 40 HFS reversed the feedforward excess to feedback of unstimulated and 5 HFS conditions (Supplementary Figure 7Bb). EC out feedback reliability of centrality changed to negative with 5 or 40 HFS. While DG in and out both decreased feedback R^2^ centrality for 40 HFS, there was little change in Cohen's *d* directionality effect (Supplementary Figure 7Bb). DG out feedback and feed-forward reliability decreased for 40 HFS but remained in the negative excess of feedback over feed-forward (Supplementary Figure 7Bb). CA3-in and CA3-out maintained their excess negative R^2^ centrality. CA1-in increased in reliability importance for feedback 5 HFS and flipped to excess negative R^2^ centrality. CA1-out remained unchanged by stimulation and effect size excess of feedback over feed-forward. In general, after stimulation, mean effect sizes favored feedback over feed-forward reliability of centrality for 12 out of 16 post-stimulation sub-region directions even though in Supplementary Figure 7B feed-forward reliability increased more than feedback reliability.

Together, these results indicated a routing strategy after stimulation that decreased the number of edges, number of degrees, increased reliability, and shifted importance toward feedback activity. This suggested that stimulation caused inhibitory feedback activity to be redirected from the routing of feed-forward activity and “sharpened” its activity for more selectivity and reliability. Feedback had more degrees than feed-forward activity coupled with increased feedback slopes over feed-forward activity made the feedback activity more central and important to the entire network. Feed-forward activity was routed through fewer routes and more reliably than the feedback activity. An increase in generalized feedback activity appeared to create more directed feed-forward activity in order to focus or sharpen the routing of information.

## Discussion

### Features summary

Communicating axons were successfully isolated in microfluidic tunnels and their response dynamics to two forms of patterned stimulus were analyzed in a full-loop hippocampal model system. The direction of communication in individual axons was discerned by spike lags between two electrodes that span the tunnels. This novel advancement of self-wiring through tunnels uniquely allowed differences in axonal spike dynamics to be measured by sub-region in the context of the overall network activity. Previously, connectivity could only be inferred by the changes in the somal activity of other sub-regions. Our reconstruction of the hippocampus allowed for the recording of the EC, DG, CA3, and CA1 sub-regions of the trisynaptic loop while simultaneously measuring the information flow between sub-regions in the axons in context with all sub-regional activity.

Spiking dynamics in our system displayed canonical non-Gaussian distributions inherent in the computations of neural systems (Beggs and Timme, 2012). All sub-regions and axonal tunnels showed linear log–log distributions of spike intervals, burst intervals, and spikes per burst. Intraburst interval distributions suggested an up-and-down state of network computation previously reported by Vakilna et al. (2021). Burst durations and intraburst spike rates were log-normally distributed. With stimulation, all interspike intervals and the number of spikes decreased from no stimulation in both soma and axons in the feed-forward and feedback directions, consistent with the Figure 1C hypothesis of decreased activity while maintaining network balance. In sub-regions, EC and CA1 had similar ISI slopes in response to the same stimulus and DG and CA3 slopes were also similar. After stimulation, EC-DG and CA3-CA1 axon directional activity remained at their previous feed-forward to feedback balance, while DG-CA3 and CA1-EC increased feedback activity in proportion to feed-forward activity. IBI fast bursting excitatory inputs dramatically decreased after stimulation by up to 87% for EC-DG, DG-CA3, and EC-CA1, until processing in CA3-CA1 which had an increase in feed-forward fast bursting. All burst durations in soma and axons decreased after stimulation, and all intraburst spike rates increased. It is important to note that after stimulation, not all changes were monotonic with increased stimulation. Instead, dynamics are balanced within a small range after stimulation, which suggests different E/I balance and computation with different stimuli.

Functional connectivity analysis between sub-region neurons and axons in tunnels revealed a refinement in routing and a sharpening of network computation with stimulation. By graph analysis, there was a decrease in the average number of degrees (functional connections) per electrode in the feed-forward and feedback DG, feed-forward and feedback CA3, and feed-forward CA1. This was coupled with an increase in the reliability of spike rate correlations between soma and axon in both directions in the EC, feed-forward DG, both directions in the CA3, and both directions in the CA1. Effect size calculations indicated that node degrees were weighted toward the feedback direction with only the EC-in no stim and 5 HFS effect sizes showed a preference for the feed-forward and EC-in 40 HFS showed no significant effect size in either direction. These connections revealed few changes in well-tunnel and tunnel-well spike rate correlations comparing before and after stimulation. These factors combined suggest that the network responded to stimulation by balanced excitatory and inhibitory activity, not with increases in directional stimulation, but with directed inhibitory behavior that modulated the routing of feed-forward computations as in model Figure 1C.

### More inter-regional feedback activity than expected for the feedforward trisynaptic network

Our graphic analysis based on the directionality of inter-regional communicating axons indicated substantial feedback routing that likely protects the network from runaway excitatory exhaustion. Inhibitory interneurons have extensive branching locally in each sub-region for local control despite comprising only 10–15% of the cells in each sub-region (Pelkey et al., 2017). GABAergic control is activated through either feed-forward (Lawrence et al., 2003) or feedback directions (Pelkey et al., 2017) to limit burst durations. For example, feedback from the CA3 to the DG hilar neurons disynaptically controls the “detonation” of DG neurons (Lawrence et al., 2003; Scharfman, 2007). The extent of inhibitory axonal sub-regions is not clear (Penttonen et al., 1997) and the only knowledge we have of comparing feedback between sub-regions comes from our last publication (Vakilna et al., 2021). Reciprocal connections have been reported between the EC and CA1 (Rockland and Van Hoesen, 1999) and CA3-DG (Penttonen et al., 1997; Lisman, 1999). CA1–CA3 inhibitory connections are believed not to exist (Schultz and Engelhardt, 2014), but they have been reported in slice stimulation experiments (Andersen et al., 2000). Our analysis shows inhibition of varying degrees from all sub-regions, which indicates that inhibition between sub-regions is possible and important for modulating information flow.

Evidence for the inhibitory nature of feedback routing was not directly available from our measures of spike dynamics. However, bicuculline can be used as a GABAA antagonist to decrease the amount of inhibitory activity that is present. If the network has feedback connections that truly are inhibitory, then we should see an almost immediate increase in feedforward axonal activity after the addition of bicuculline as we have seen in single-compartment cultures (Khatami et al., 2004; Boehler et al., 2007).

Perhaps because Lorente de No originally described the hippocampus in terms of largely feed-forward architecture, reports of inter-regional feedback axons are few. Our networks show a large amount of feedback activity between all sub-regions, which differs from the canonical representations of the trisynaptic loop. For example, our networks show feedback activity from the DG to the EC, which has not been described in the literature. However, the Allen Brain Atlas shows experiments with a marker placed in the DG resulting in labeled axonal projections back to the first, second, and third layers of the EC (Experiment 112745787–DG). These connections are few, but from a control systems perspective, are necessary to modulate the flow of excitatory information.

### Overall E/I balance dynamics

A synthesis of this information supports hypothesis C in Figure 1. Figures 8C, D shows a sub-regional summary of the change in degrees and reliability. Stimulation caused the network to route functional connections through fewer paths, except for EC feed-forward (summarized in Figure 7B). The remaining functional connections were more reliable in 12 of the 16 centrality measures (R^2^, Figure 8B), particularly in CA3 neurons that feed forward through axons into CA1. This routing strategy in response to electrical stimulation is influenced by the overall E/I balance of the spiking dynamics and routing. Unlike computer circuitry, the circuits of the brain do not evoke the same response to the same input every time. This is a feature of the spatial dynamics of the system that reflect the probabilities of network plasticity.

The coding for information transmitted between hippocampal sub-regions likely comes in bursts of action potentials. Bursts in neural networks may be analogous to packets in Internet communication: The information is not sent all at once but is instead chopped up and recombined or interpreted at a specified address, which improves the reliability of message transmission (Graham and Rockmore, 2011). In cortical circuits, these bursting packets are proposed to comprise the building blocks of information transmission. Based on the number and exact timing of spikes within bursts and the timing of the bursts, they convey information about the type of stimulus, a form of pattern recognition, as we have identified between the DG and CA3 (Bhattacharya et al., 2016). As a control in a two-compartment system, Brewer et al. (2013) found specific aspects of spike dynamics in DG-CA3 compared with DG-DG and CA3-CA3, suggesting that appropriate anatomy produced distinct coding. Structured spontaneous activity has a similar firing pattern to the evoked stimulus and may be a replay of the stimulated signal. Hence, current activity is most similar to the response to recent stimuli up to several minutes after exposure (Luczak et al., 2015). The hippocampus organizes spikes into 50–200 ms packets (Luczak et al., 2015), similar to the 40–100 ms median burst lengths we observed. Our graphical analysis combined with slow burst rates indicated rerouting with stimulation that would be the basis for predictive learning while minimizing energy expenditure for maximum computational efficiency (Luczak et al., 2022).

The balance and modulation of E/I activity in spontaneous and stimulated conditions allow for efficient neural encoding of memories (Zhou and Yu, 2018). How spontaneously firing neurons establish their irregular firing patterns could be key to understanding network states and the representation of stimulus inputs. To achieve global E/I balance, the neurons must be sparsely connected at random, and the strength of the inhibitory connections should be high enough to control runaway excitation (Zhou and Yu, 2018). The live networks we have cultured meet these criteria through a lack of one-to-one connections in functional connectivity analysis and an abundance of feedback activity causing lowered spike rates after stimulation.

Pyramidal neurons typically maintain a tight range of firing rates for optimal information processing, but inhibitory inter-neurons respond over a wider dynamic range (Maffei and Fontanini, 2009). There is also evidence for inhibitory interneurons not only controlling local homeostasis but also coordination across sub-regions to maintain higher order stability (Maffei and Fontanini, 2009). These feedback connections provide a control mechanism for keeping networks from runaway metabolic exhaustion. Hippocampal inhibitory interneurons only account for 10–15% of the total cell population, so their ability to keep the network balanced with such a low percentage of the total input is based on their highly branched topology and the types of interactions they form for overall global balance (Pelkey et al., 2017; Kajiwara et al., 2021). An additional, largely underexplored possibility that we investigated here was the ability of inter-regional axons to impose feedback inhibition onto the prior sub-region in the network. How these mechanics balance globally and locally in the hippocampus can provide information on network computational functions.

In previous reconstructions of the hippocampal DG to CA3 sub-regions through axonal tunnels, we found activity dynamics were composed of spontaneous repeating spatio-temporal motifs and that differences in first-to-fire probabilities depended on tunnel routes (Bhattacharya et al., 2016; Poli et al., 2018). With paired-pulse stimulation in engineered pairs of hippocampal sub-regions, we found the strongest evidence for sparse coding and pattern separation in the DG and for pattern completion in the CA3. In the current report of reconstruction of the entire trisynaptic loop, stimulation evoked functional pruning of the tunnel connections and prioritized selected routes in the network, which can be considered a plastic event. This is important because this changes which cell is first to fire also known as the leader neurons (Ham et al., 2008) and the downstream firing patterns of spikes and bursts (Eckmann et al., 2008).

We have already described in detail the changes to firing patterns of spikes and bursts, but we have yet to study the effects of stimulation on probabilities of first-to-fire neurons. We previously exposed cultured networks to high-frequency stimulation over hour-long intervals while the cells were still developing which generally led to higher spike rates and larger functional networks (Brewer et al., 2009a; Leondopulos et al., 2012). Exposing the network to high-frequency stimulation at theta frequency repeats over only a few minutes when the network was fully grown conversely lowered the spiking and burst rates. In the graph analysis we conducted, the DG and CA3 axons were the most plastic sub-regions as the number of functional connections changed more than other sub-regions. The DG particularly had the most plasticity as stimulation decreased the number of functional connections and therefore sharpened routing in both directions of its outgoing axons. As a result, the CA3 feed-forward outgoing axons greatly decreased the number of degrees. Considering the CA3 has the smallest overall axonal and sub-regional change in spike rates and burst rates yet the largest variation in a number of functional connections, this may be a balancing mechanism to maintain a narrow range of functional operations. DG mossy fiber inputs to the CA3 are responsible for the plasticity of networks and encoding of memory (Kesner and Rolls, 2015), so seeing the most plasticity in these regions is consistent with studies *in vivo* (Kesner and Rolls, 2015). In a reconstructed hippocampus, this could mean that the network is encoding “memory-like” information in the CA3 along a narrower range of circuits from homeostatic conditions. However, more in-depth temporal analyses are necessary to make a stronger conclusion.

### Narrow window of spiking dynamics post-stimulation

After stimulation, the network dynamics stayed within a narrow range. ISI slopes decreased after stimulation in both the sub-regions and the axons. Only EC-DG feed-forward and feedback, CA3-CA1 feed-forward, and EC-CA1 feedback axons showed a significant change in ISI between the two stimulation cases at less than a 20% difference between them. Similarly, bursting intervals, burst duration, and intraburst spike rate remained similar in a majority of sub-regions after stimulation. In the wells, only the fast-bursting CA1 cells had a significant difference of 31% between HSF 5 and HFS 40 stimulations. Half the tunnels, feed-forward EC-DG, DG-CA3, and CA3-CA1, and feedback CA3-CA1 showed significant changes by ANOVA. The magnitude of these percentages is between 7 and 49%. However, these percentage differences in burst rate are so large because the slopes of some of these bursts are so shallow. These narrow ranges may relate to optimality in neural networks. At rest, a network can still be active and not be computing any information for the sake of inertia, like an idling automobile. When the same network responds to a stimulus for computing information, it operates over a narrow dynamic range around an optimal point and is highly sensitive to changes within that range (Beggs, 2022). As our measurements were taken 30–60 min after the stimulation, our reported network dynamics reflect enduring plasticity in response to the stimulus. Investigations into the classical criticality of these networks after stimulation are ongoing.

### Limitations

Our *in vitro* model network may not reflect *in vivo* spike dynamics for several reasons. Our network was cultured in two dimensions which greatly simplifies the complexity of the network in comparison with the 3D axonal organization *in vivo*, which would enable multilevel signal integration (Jensen and Teng, 2020). However, much of the functional connectivity of the hippocampus occurs in a narrow 200–400 μm thickness. The principal cells in sub-regions of the *in vivo* hippocampus are laminated, while our model neurons are dispersed. This likely interferes with the layered axonal innervation of proximal vs. distal dendrites. *In vitro* 3D cultures overall respond more robustly to stimuli and are more relevant to *in vivo* systems (Frega et al., 2014; Antoni et al., 2015; Tedesco et al., 2018). Yet these 3D cultures have not yet isolated axons as we have.

Our laboratory is currently working on solutions to culturing hippocampal cells in 3D with electrophysiological monitoring. Additionally, there are thousands of neurons per sub-regional chamber, yet in each recording only approximately 19 could be recorded from in each sub-region. Similarly, 51 axon tunnels connect each sub-region and electrodes only span 5 of those interregional axon tunnels. These factors combine to limit our graph analysis to functional connections between some axons in tunnels and some soma in wells.

There are anatomical factors in our reconstructed hippocampi that do not appear *in vivo*. Firstly, this is an isolated system with no inputs from upstream brain regions such as the neocortex, parahippocampal gyrus, medial septum, or perirhinal cortex. Our reconstructed networks are missing the perforant pathway from the EC to the CA3; however, we have constructed MEMS systems that will allow for this pathway to self-wire (Chen et al., 2023). Additionally, the subiculum and the EC dissections were not separated, and the multiple layers of the EC were included in one EC sub-region. In our networks, we have feedback activity from the EC to the CA1 and feedback activity from the DG to the EC. We cannot tell whether these connections are inhibitory or excitatory without bicuculline studies.

Only the spontaneous activity of the networks was recorded after stimulation trains were delivered. Stimulation artifacts precluded reading from electrodes during stimulation. Therefore, we did not assess the instantaneous response of the network to stimulation and the factors of Hebbian synaptic plasticity that influence reliable plasticity or learning. We recently reported plasticity responses to patterned stimulation within 10 ms of the stimulus (Chen et al., 2023). However, we see network activity changes 67 ± 20 min (SD) after stimulation, which indicates that LTP and LTD have occurred. *In vivo* inputs are likely to stimulate more than the three neurons we stimulated in this model. Defined multicell patterned stimulation through optogenetics is being developed in our laboratory and others (Barral and Reyes, 2017).

### Future study

Now that we have evaluated the spatial spiking dynamics underpinning hippocampal network architecture, it is important to look at other computational mechanisms of the network and what that means for the type of computations the network is performing. There has been a long-standing debate about what oscillatory signals mean in the brain in relation to spikes. The synchronization between spikes and oscillations between 1 and 100 Hz needs to be studied on multiple spatiotemporal scales to determine whether they are independent computational functions or work in tandem with spikes. Additionally, the network can be further categorized by its states of computational activity. Criticality is the idea that the brain has an optimal dynamic range for computations (Beggs, 2022). To determine what narrow dynamic ranges are optimal for our cultured networks, criticality measurements will be applied.

With the emergence of consumer-grade spiking neural network hardware, it is time to start applying biologically inspired algorithms where classical artificial neural network algorithms have provided no advantage. We have the ability to directly study hippocampal network behavior and can apply those algorithms and architectures to complement spiking neural network hardware. Once the oscillatory behavior and optimal ranges of computation are better understood, neuromorphic software and hardware architecture can be constructed with better graph algorithms inherent to hippocampal computation (Davies et al., 2021).

## Conclusion

Overall interregional network activity is controlled by an abundance of feedback axons between all sub-regions in the context of the trisynaptic loop beyond the widely appreciated intraregional inhibitory interneurons. Differential spiking from four sub-regions of the hippocampal formation and their communicating axons suggested functional differences in information processing in response to stimulation. Stimulation specifically altered the log–log distributions of spike dynamics for inter-spike intervals, inter-burst intervals, burst duration, intra-burst spike rate, and spikes per burst. Generally, firing rates decreased proportionally after stimulation; feedforward and feedback spiking selectively decreased with stimulation and the dynamics were affected by a small excess of axonal feedback. Additionally, the number of functional connection routes decreased (as in Figures 1C, 8C, D), but the reliability of those connections increased in a phenomenon we call network sharpening. The sharpening effect was particularly strong in CA3 feed-forward axons into CA1.

## Data availability statement

Our data is available here: https://datadryad.org/stash/dataset/doi:10.5061/dryad.7h44j1013 and our code is available here: https://github.com/Brewer-Neurolab/Lassers-2023-Axon-Flow.

## Ethics statement

The animal study was approved by UC Irvine Institutional Animal Care and Use Committee. The study was conducted in accordance with the local legislation and institutional requirements.

## Author contributions

SL: Data curation, Formal analysis, Investigation, Software, Validation, Visualization, Writing—original draft, Writing—review & editing. YV: Data curation, Software, Visualization, Writing—review & editing. WT: Supervision, Validation, Writing—review & editing. GB: Conceptualization, Data curation, Funding acquisition, Investigation, Methodology, Project administration, Resources, Supervision, Validation, Writing—original draft, Writing—review & editing.

## Supplementary material

Supplementary Figure 1 Peaks in conduction time histograms allow identification of spike directionality and corresponding wave forms for axon unit identification. Red bars are spike pairs at peak above threshold (horizontal line) ± two bins. Blue bars are spike pairs below threshold. **(A)** Feed-forward peak (red) and discarded low activity peak (blue), with corresponding waveforms on each electrode. **(B)** Two feed-forward peaks, U1 AND U2. **(C)** Single feed-forward peak identified above background noise. **(D)** Bidirectional axons in a single tunnel identified.

Supplementary Figure 2 ISI slopes comparing activity between subregions within a stimulation series for wells, feed-forward, and feedback. Comparisons between subregions show distinct subregional and directional computation meaning the networks are not homologous in cellular make up or computation. Almost all slopes were different unless otherwise stated in the figure. **(A–C)** Well ISI before and after stimulation. **(D–F)** Feed-forward axon ISI before and after stimulation. Feed-forward ISI follows well ISI showing that the whole network is connected. **(G–I)** Feedback axon ISI before and after stimulation. Feedback axon ISI are higher than the feed-forward ISI.

Supplementary Figure 3 Normalized fractional slow vs fast bursting (fast-slow)/(fast+slow)*100 show a decrease from fast bursting (blue bars before stimulus) toward slow bursting in most soma and axons after stimulus, suggesting network balancing toward slow bursting after stimulation. **(A)** shows a shift toward slow bursting slopes in DG. For feed-forward axons, **(B)** shows a shift away from fast bursting in EC-DG, DG-CA3, and CA1-EC. The largest change again is DG-CA3. There was a shift toward fast bursting in CA3-CA1. For feedback axons, C shows a shift away from fast bursting in EC-DG and CA3-CA1. There was no change in DG-CA3 feedback axons, but there was a shift toward 0 in CA1-EC feedback axons. Feedback axons largely balanced the network toward zero bursting more than the well and feed-forward bursting.

Supplementary Figure 4 Five HFS and 40 HFS generally shorten the burst duration compared to no stimulation. Semi-log probability distributions represented as red dashed lines with a single Gaussian fit, median peak, correlation coefficient, and fits over 200 logarithmically spaced bins. The vertical blue line represents the no stimulation median. **(A)** Well electrode burst duration decreased from the no stimulation sequence. **(B)** Feed-forward axon burst duration medians decreased 8 of 8 cases after stimulation. **(C)** Feedback axon burst duration decreased from no stimulation in 8 of 8 cases after stimulation.

Supplementary Figure 5 Intra burst spike rates followed a semi log probability distribution represented as red dashed lines with a single Gaussian fit. Correlation coefficients and medians of the distribution are reported. The vertical line represents the no stimulation median. **(A)** Well electrode Intra burst spike rate fits all showed an increase from the no stimulation sequence. **(B)** Feed-forward axon Intra burst spike rate fits increased from no stimulation 8 of 8 times after stimulation. **(C)** Feedback axon Intra burst spike rate fits decreases from no stim 7 of 8 times after stimulation.

Supplementary Figure 6 Spike frequency (number of spikes/100 ms, average firing rate, AFR, Hz) regressions in 100 ms bins for 10 s windows categorizing functional connections between tunnels and axons. Out of 100 possible points (100 0.1 s bins in 10 s), those with spikes are plotted. Many bins had no spikes. Slopes greater than one are excitatory and slopes less than one are inhibitory. **(A)** Edge weights for slopes and R^2^ are plotted as averages for windows with unique values. **(B–D)** Feed-forward (FF) and feedback (FB) functional connection between well-tunnel and tunnel-well MEA-120 electrodes; directionality between electrode names is denoted alphanumerically (pin layout). **(B)** High slopes and high R^2^ are excitatory when comparing to the average. **(C)** Low slopes and high R^2^ are inhibitory when comparing to the average. **(D)** Poor slope and poor R^2^ are included in averages if above thresholds and suggest network was not currently routing through a specific tunnel.

Supplementary Figure 7 The importance of nodes to functional connectivity did not change with stimulation. But the network assigned more importance to feedback connections than feed-forward connections. **(A)** Slope centrality is a weighted degree measurement which represents the importance of the source node's connections and evoked spike rate in the target channel. Slope centrality decreases from no stimulation only in the DG-In, CA3-In, and CA3-Out cases. Overall effect size is toward feedback importance. **(B)** R^2^ centrality increases in feedback EC-In, EC-Out and CA1-In. The importance of the reliability increased in EC-In, EC-out, and CA1-In feedback and decreased in DG-In and DG-out feedback and DG out feed-forward. Overall effect size is toward feedback importance for reliable connections.

Supplementary Table 1 Spontaneous recordings and timing.
