# Supplementary figures and images for: The flow of axonal information among hippocampal sub-regions 2: patterned stimulation sharpens routing of information transmission
Source: Front Neural Circuits. 2023 Dec 8;17:1272925. doi: 10.3389/fncir.2023.1272925 (PMC10739322; doi:10.3389/fncir.2023.1272925)

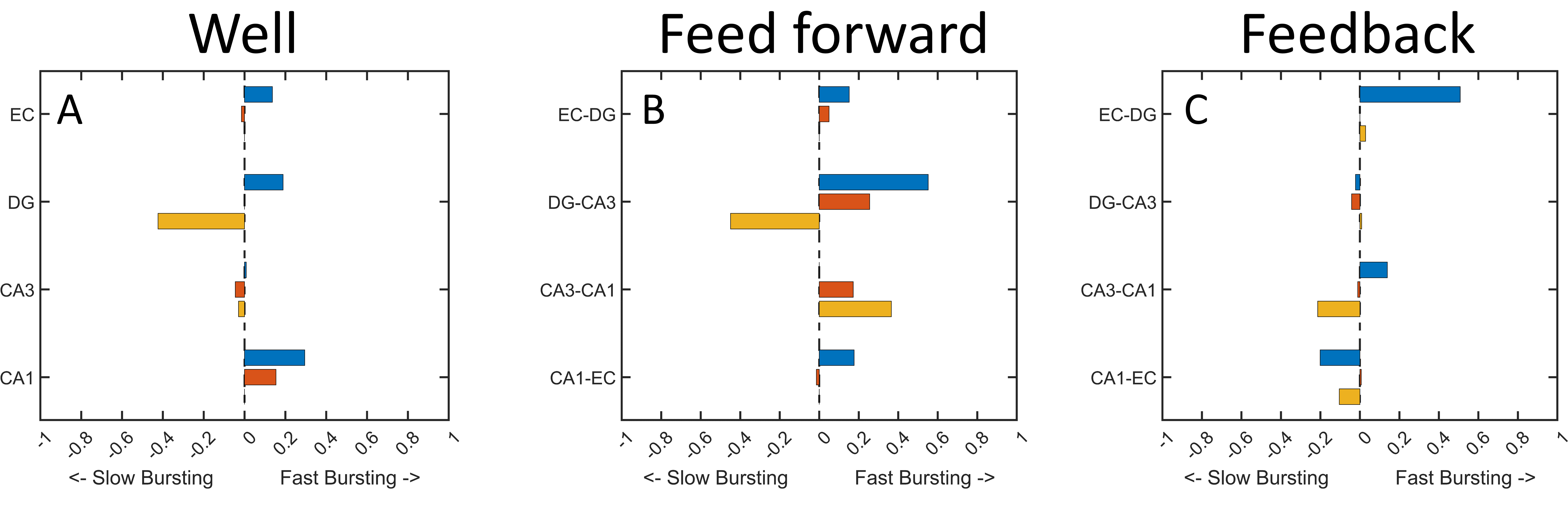

Supplement: Supplementary Figure 3 — Normalized fractional slow vs fast bursting (fast-slow)/(fast+slow)*100 show a decrease from fast bursting (blue bars before stimulus) toward slow bursting in most soma and axons after stimulus, suggesting network balancing toward slow bursting after stimulation. (A) shows a shift toward slow bursting slopes in DG. For feed-forward axons, (B) shows a shift away from fast bursting in EC-DG, DG-CA3, and CA1-EC. The largest change again is DG-CA3. There was a shift toward fast bursting in CA3-CA1. For feedback axons, C shows a shift away from fast bursting in EC-DG and CA3-CA1. There was no change in DG-CA3 feedback axons, but there was a shift toward 0 in CA1-EC feedback axons. Feedback axons largely balanced the network toward zero bursting more than the well and feed-forward bursting. [file Image_3.TIF]

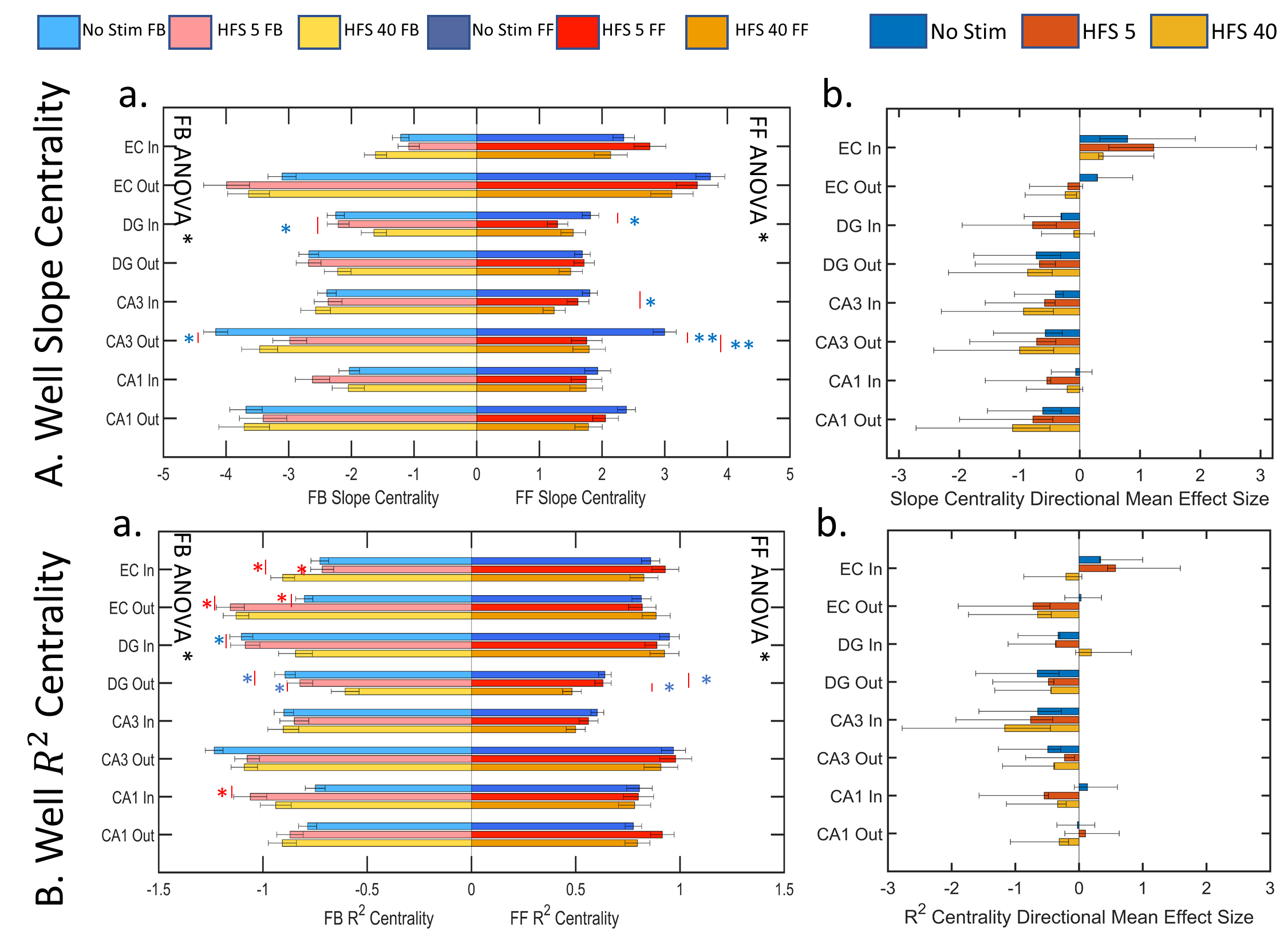

Supplement: Supplementary Figure 7 — The importance of nodes to functional connectivity did not change with stimulation. But the network assigned more importance to feedback connections than feed-forward connections. (A) Slope centrality is a weighted degree measurement which represents the importance of the source node's connections and evoked spike rate in the target channel. Slope centrality decreases from no stimulation only in the DG-In, CA3-In, and CA3-Out cases. Overall effect size is toward feedback importance. (B) R2 centrality increases in feedback EC-In, EC-Out and CA1-In. The importance of the reliability increased in EC-In, EC-out, and CA1-In feedback and decreased in DG-In and DG-out feedback and DG out feed-forward. Overall effect size is toward feedback importance for reliable connections. [file Image_7.TIF]
